# Three Component One-Pot Synthesis and Antiproliferative Activity of New [1,2,4]Triazolo[4,3-*a*]pyrimidines

**DOI:** 10.3390/molecules28093917

**Published:** 2023-05-05

**Authors:** Manel Ben Hassen, Dhouha Msalbi, Badr Jismy, Fares Elghali, Sami Aifa, Hassan Allouchi, Mohamed Abarbri, Fakher Chabchoub

**Affiliations:** 1 Laboratory of Applied Chemistry: Heterocycles, Lipids, and Polymers, Faculty of Sciences of Sfax, University of Sfax, Sfax 3000, Tunisia; 2 Laboratory of Molecular and Cellular Screening Processes, Centre of Biotechnology of Sfax, Sfax 3018, Tunisia; 3 Laboratory of Physico-Chemistry of Materials and Electrolytes for Energy (PCM2E), Faculty of Science and Technology, University of Tours, 37200 Tours, France; 4 Faculty of Pharmacy, University of Tours, 37200 Tours, France

**Keywords:** multicomponent reaction, one-pot reaction, triazolopyrimidine, breast cancer, antitumoral

## Abstract

A series of new [1,2,4]triazolo[4,3-a]pyrimidine derivatives was prepared using a one-pot three-component synthesis from 5-amino-1-phenyl-1*H*-1,2,4-triazoles, aromatic aldehydes and ethyl acetoacetate. The compound structures were confirmed by IR, ^1^H-NMR, ^13^C-NMR, HRMS and X-ray analyses. The biological activity of these compounds as antitumor agents was evaluated. Their antitumor activities against cancer cell lines (MDA-MB-231 and MCF-7) were tested by the MTT in vitro method. Among them, compounds **4c** and **4j** displayed the best antitumor activity with IC_50_ values of 17.83 μM and 19.73 μM against MDA-MB-231 and MCF-7 cell lines, respectively, compared to the Cisplatin reference.

## 1. Introduction

Heterocyclic compounds have received special attention in organic chemistry due to their presence in many natural products and their diverse biological properties [1]. Among them, pyrimidines and their derivatives occupy an important place due to their biological importance for medicinal chemistry [2,3,4].

**Figure 1:**
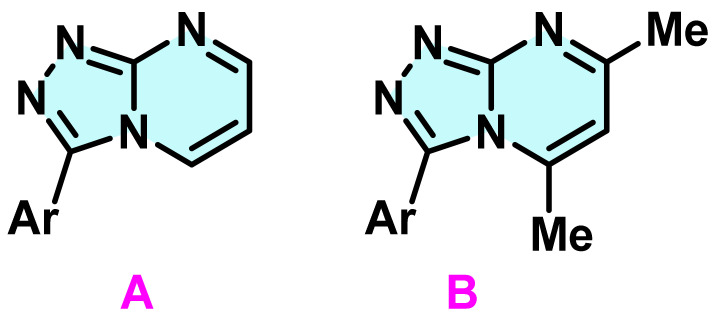
Some examples of biologically active [1,2,4]triazolo[4,3-*a*]pyrimidines.

A review of the literature also revealed that substituted 1,2,4-triazoles and their bridged heterocyclic derivatives have attracted particular attention over the last two decades due to their wide range of therapeutic properties [5]. Fusion of the 1,2,4-triazole ring with the pyrimidine ring gives rise to the formation of bicyclic heterocycles called 1,2,4-triazolopyrimidines (TPs), where the common nitrogen of the triazole and pyrimidine occupy the ring junction. These triazole-fused pyrimidines have been the subject of increasing interest for their various important pharmaceutical properties [6,7,8]. They appear in many synthetic pharmacophores that possess antiparasitic, antimicrobial, anticancer and antibiotic activities [9,10,11]. In particular, many triazolo[4,3-*a*]pyrimidines exhibited antiproliferative effects against HePG-2 and MCF-72a cell lines [12] and cytotoxic activity on a human hepatic carcinoma cell line (HEPG2) [13]. For example, 1,2,4-triazolo[4,3-*a*]pyrimidines **A** and **B** were reported to show antibacterial activity [14,15], and compound **B** showed higher DNA photocleavage activity [16] and has been described as a potent apoptotic inducer [17] (Figure 1).

Note also that the use of triazolopyrimidines in drug design has recently been reported [18]. In addition, this class of compounds has also been of great importance in the field of agriculture due to their remarkable activities as herbicides [19]. Consequently, TPs have inspired excellent reviews in the literature over the years on their chemistry and synthetic methods [20,21,22,23,24].

Despite the many syntheses described in the literature of 1,2,4-triazolo[1,5-*a*]pyrimidines, only a limited number of syntheses of [1,2,4]triazolo[4,3-*a*]pyrimidines have been reported. Generally, the most commonly used syntheses are cyclization of 3-ethoxycarbonyl-2-hydrazinylpyrimidines [25]; or by reactions of 2-hydrazinylpyrimidines with different reagents such as benzoyl chloride, CS2, formic acid [12], acetic anhydride, ethyl chloroformate and triethyl orthoformate [13]; or by oxidation of 2-(2-benzylidenehydrazinyl)-pyrimidines with Br_2_ in AcOH [26]; or by the reaction of 3,4-dihydropyrimidine-2(1*H*)-thiones with ethyl 2-(arylhydrazinylidene)-2-haloacetate [27]. However, the reported methods generally suffer from some drawbacks, such as harsh reaction conditions and little flexibility offered. Therefore, the development of an efficient and economical method for the synthesis of [1,2,4]triazolo[4,3-*a*]pyrimidines involving available starting materials is highly desired.

In the continuity of the syntheses developed within our group using condensed pyrazoles, which have proven to be of biological interest [28,29,30,31], we report herein the three-component synthesis of [1,2,4]triazolo[4,3-*a*]pyrimidines in a one-pot manner and preliminary results for in vitro antiproliferative evaluation. Due to their flexibility to assemble several reagents in a short time and transform them into compounds of interest in a one-pot procedure [32,33], multi-component reactions (MCRs) have become very popular in the development of new biologically active compounds. Additionally, MCRs are favored for their experimental simplicity, their atom-/step economy, their reduced reaction time and the high yields produced compared to classical chemistry [34]. Note that syntheses of some condensed triazolopyrimidine derivatives using multicomponent one-pot methods have also been reported [35,36].

## 2. Results and Discussion

### 2.1. Chemistry

In our initial investigation, we sought to optimize the conditions for this multicomponent reaction using aminotriazole **1a**, benzaldehyde **2a**, and ethyl acetoacetate as starting reagents for the reaction model, by varying the catalyst and the solvent (Table 1).

In our first attempts, we performed the reaction using 10 mol% of *para*toluene sulphonic acid (APTS) as a catalyst and H_2_O as a solvent under reflux for 24 h, but only traces of the desired product **4a** were observed (Table 1, entry 1). Interestingly, when water was replaced by ethanol at reflux, the desired product **4a** was isolated in 75% yield (Table 1, entry 2). Under the same conditions but replacing APTS with HCl, the expected product **4a** was formed in a low yield (45%) (Table 1, entry 3). This decrease in yield was even more pronounced when acetic acid was used as a catalyst (Table 1, entry 4). The use of a basic catalyst such as piperidine proved unsuccessful (Table 1, entry 5). In the absence of any solvent and catalyst, and by heating until the reaction mixture had completely melted, the desired product **4a** was isolated with a yield not exceeding 10% (Table 1, entry 7). A reduction in the reaction time to 18 h at the reflux of ethanol in the presence of 10 mol% of APTS resulted in an incomplete conversion rate. The use of a polar aprotic solvent such as acetonitrile did not give complete satisfaction since the yield of the isolated product **4a** was only 4 50% (Table 1, entry 6). Consequently, following the results recorded in Table 1, the best conditions for obtaining compound **4a** with the best yield were the use of APTS as a catalyst (10 mol%) at the reflux of ethanol for 24 h (Table 1, entry 2).

**Scheme 1:**
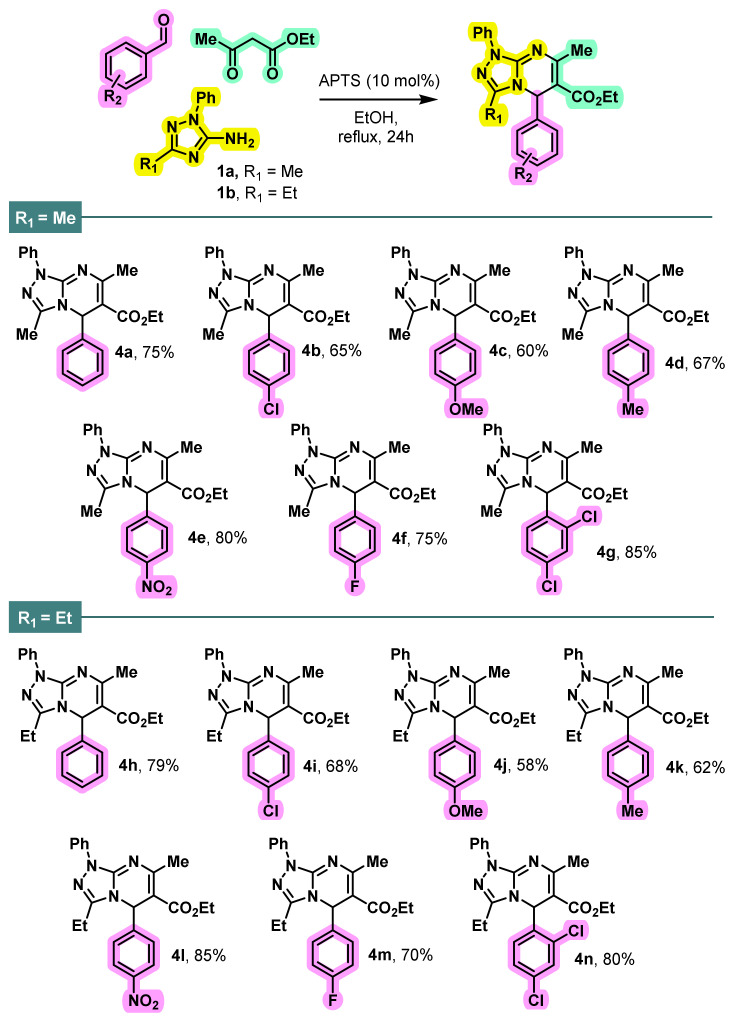
Synthesis of multi-substituted [1,2,4]triazolo[4,3-*a*]pyrimidines **4a**–**n**.

Based on the optimized reaction conditions, a series of new ethyl multisubstituted-1,5-dihydro-[1,2,4]triazolo[4,3-*a*]pyrimidine-6-carboxylates **4a**–**n** were therefore synthesized using 5-amino-1-phenyl-1*H*-1,2,4-triazoles **1a**–**b,** aromatic aldehydes **2a**–**g** and ethyl acetoacetate **3** (Scheme 1).

As illustrated in Scheme 1, the three-component reaction is compatible with a wide range of aromatic aldehydes containing electron donating or electron withdrawing substituents. Indeed, aldehydes carrying electron donor groups such as CH_3_, C_2_H_5_ or electron withdrawing groups such as Cl and F were easily condensed on 5-aminotriazoles **1a**–**b** in the presence of ethyl acetoacetate, providing access directly to the desired compounds in good to excellent yields. In addition, this reaction was also compatible with aldehydes substituted by a strongly electron-donating group such as OCH_3_ or a strongly electron-withdrawing group such as NO_2_. Likewise, the use of an aldehyde substituted by two chlorine atoms on the aromatic ring successfully led to the desired products **4g** and **4n** with respective yields of 85 and 80%.

As also observed, the efficiency of this multicomponent reaction was not affected by the nature of these substituents. Similarly, there was no significant difference in the yields of the reactions depending on the nature of the substituents, either on the aminotriazole or on the aromatic aldehyde.

**Scheme 2:**
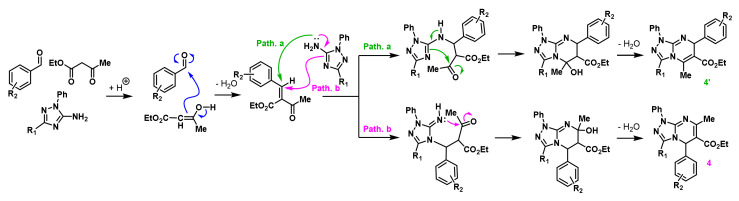
Plausible mechanism for the formation of [1,2,4]triazolo[4,3-*a*]pyrimidines **4a**–**n**.

Based on the above results and drawing inspiration from the results reported in the literature [37,38], we hypothesized that the mechanism of the three-component one-pot reaction could be as follows (Scheme 2).

The reaction begins with an attack of the pi doublet of the enol form of ethyl acetoacetate **3** on the carbonyl of the aromatic aldehyde leading subsequently, after the elimination of a molecule of water, to the corresponding arylidene according to a known Knoevenagel-type reaction. After that, the reaction can proceed along two different pathways:

In pathway a, there is a 1,4-Michael-type addition of a doublet of the amino group of aminotriazole **1** on the most electrophilic carbon of the arylidene, leading to a reaction intermediate, which undergoes intramolecular lactamization followed by dehydration to lead to compound 4′.

In pathway b, it is the doublet of nitrogen 4 of 5-amino-1-phenyl-1*H*-1,2,4-triazole **1** that attacks the electrophilic carbon of the arylidene, which, after intramolecular cyclization followed by dehydration, leads to isomer **4**. This regioselectivity of the reaction appears logical insofar as the Michael addition involves the most nucleophilic nitrogen atom of the aminotriazole **1**.

To elucidate the real structures of the synthesized compounds **4a**–**n**, we carried out a 2D NMR HMBC sequence on compound **4f,** since one-dimensional NMR does not make it possible to decide between these two isomers of position 4′ and 4. We observed on the one hand a correlation between the C3 carbon and the H5 proton, and on the other hand a correlation between C5 and the methyl group protons attached to the C7 carbon (see Figure S27 in the Supplementary Materials), thus proving the unique formation of isomer **4**.

**Figure 2:**
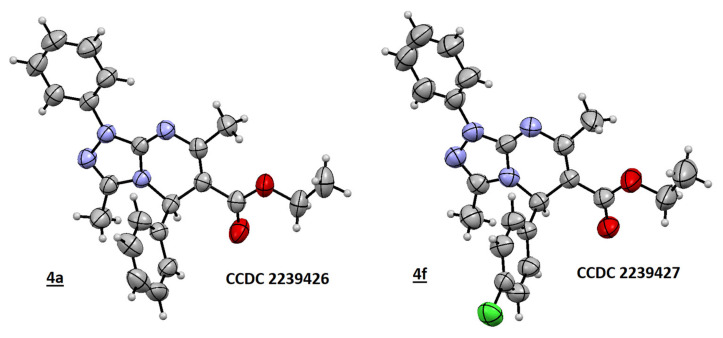
X-ray single-crystal structures of compounds **4a** and **4f**.

In addition to evidence from 2D NMR spectroscopic analysis, X-ray crystallographic analyses of single crystals of compounds **4a** and **4f** strongly confirmed the structures of the cyclo-condensation compounds **4a**–**n** (see Figure S2 and the Supplementary Materials for X-ray data of **4a** and **4f**). The ORTEP diagrams shown in Figure 2 clearly show the formation of type 4 compounds, thus unambiguously confirming the high regioselectivity of this reaction [39].

Next, the biological potential of the synthesized compounds **4a**–**n** as antitumor agents was subsequently evaluated.

### 2.2. Biological Activity: Anticancer Activity In Vitro

All target compounds **4a**–**n** were evaluated for their antitumor activity in vitro by the MTT method. The IC_50_ values against MDA-MB-231 and MCF-7 (human breast cancer cell lines) are summarized in Table 2.

Table 2 shows that the synthesized triazolopyrimidines **4a**–**n** exhibited inhibition of the proliferative activity of MDA-MB-231 and MCF-7 cancer cells. These compounds make it possible to improve the efficacy of the antitumor response compared with *Cis*platin, particularly as the most potent inhibitory molecules are those that have a more significant decrease in IC_50_ values. These values are 17.83 μM and 20.33 μM for compound (**4c**) bearing R = CH_3_, R_1_ = H, R_2_ = CH_3_O, and 20.97 μM and 19.73 μM for compound (**4j**) bearing R = CH_2_-CH_3_, R_1_ = H, R_2_ = CH_3_O, respectively, _P_2 times and 4 times more potent than Cisplatin (one of the most effective FDA-approved treatments for breast cancer [40]). It appears, in this case, that the presence of the CH_3_O moiety in the aryl group increases antitumoral activity.

The most potent compounds (**4c** and **4j**) will be the subject of more biological experiments leading to protein target identification and guided by docking studies in order to determine the mechanisms that induced tumor cell death. An entire investigation of cell death and the cell cycle of treated cells is in progress.

**Figure 3:**
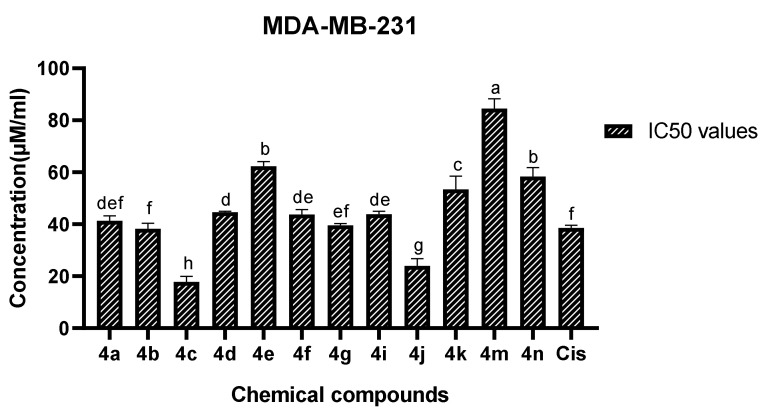
Antiproliferative activity via MDA-MB-231 cell lines.

**Figure 4:**
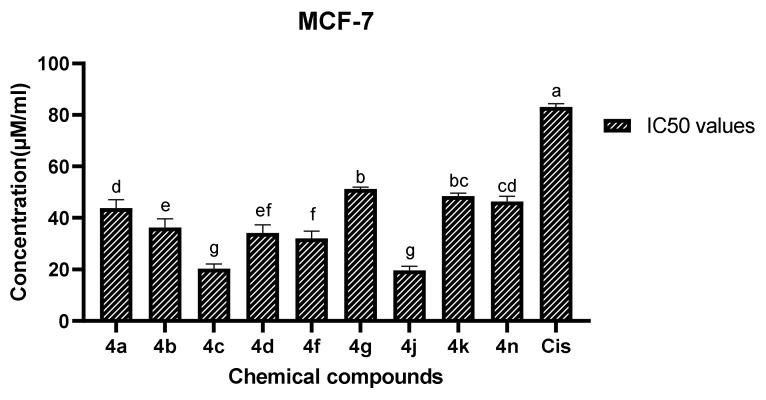
Antiproliferative activity via MCF-7 cell lines.

Figure 3 and Figure 4 present histograms showing the effect of the synthesized triazolopyrimidine compounds **4a**–**n** on the cell proliferation of the MDA-MB-231 and MCF-7 lines for 48 h.

## 3. Materials and Methods

### 3.1. Chemistry

#### 3.1.1. General Information

All thin layer chromatography (TLC) analyses were performed on type 60 F 254 silica gel n silica-gel-precoated aluminum sheets (Type60 F254, 0.25-mm thickness; from Merck, Darmstadt, Germany) with detection using a UV lamp. Melting points were determined on the Kofler and Buchner bench and were not corrected. Infrared (IR) spectra were recorded using a Perkin Elmer device whose range of precision is from 4000 to 400 cm^−1^ in powders (dispersed in a KBr pellet). All NMR spectra were recorded using a Brucker AV400 Avance spectrometer (at 400 MHz for ^1^H and 100 MHz for ^13^C). Chemical shifts are expressed in parts per million (ppm) using TMS as an internal standard in CDCl_3_.

5-Amino-1-phenyl-1*H*-1,2,4-triazole **1a**–**b** was prepared according to the literature [41] by gently refluxing in methanol.

All other reagents and solvents used were analytical grade.

#### 3.1.2. General Procedure for the Synthesis of [1,2,4]triazolo[4,3-*a*]pyrimidines **4a**–**n**

In a 50 mL flask, (3 mmol) of 5-amino-1-phenyl-1*H*-1,2,4-triazole **1a**–**b**, (3 × 10^−3^ mol) of aldehyde aromatic **2a**–**g** and (3 × 10^−3^ mol) of ethyl acetoacetate **3** were combined while adding (3 × 10^−4^ mol) of APTS as a catalyst in 10 mL of ethanol. The reaction was monitored by TLC with mobile phase ether/hexane (2/1), and after 24 h of heating under reflux, the mixture was left at room temperature. The solid that forms was filtered, washed and then recrystallized with an ethanol/ether mixture.

*Ethyl 3,7-dimethyl-1,5-diphenyl-1,5-dihydro-[1,2,4]triazolo[4,3-a]pyrimidine-6-carboxylate* (**4a**), Yellow powder, yield: 841 mg (75%), Mp 143–145 °C; IR (KBr): ν_max_/cm^−1^ ν_C=O_: 1691, ν_C=N_: 1640. ^1^H NMR (400 MHz, CDCl_3_): δ = 1.25 (t, *J* = 7.1 Hz, 3H, CH_3_), 2.19 (s, 3H, CH_3_-C3), 2.54 (s, 3H, H10), 4.06–4.16 (m, 2H, O-CH_2_), 6.18 (s, 1H, H5), 7.30–7.34 (m, 4H), 7.38–7.40 (m, 2H), 7.48 (t, *J* = 7.9 Hz, 2H), 8.20 (d, *J* = 7.9 Hz, 2H). ^13^C NMR (100 MHz, CDCl_3_): δ = 166.6 (C=O), 159.7 (C7), 148.5 (C3), 144.8 (C8a), 142.9, 137.6, 129.0, 128.7, 128.4, 127.2, 126.1, 120.0, 97.9 (C6), 59.5 (O-CH_2_), 57.6 (C5), 25.2 (C10), 14.4 (CH_3_), 11.1 (CH_3_-C3). HRMS (ESI): *m*/*z* [M + H]^+^ Calcd for C_22_H_23_N_4_O_2_: 375.1821; Found: 375.1812.

*Ethyl5-(4-chlorophenyl)-3,7-dimethyl-1-phenyl-1,5-dihydro-[1,2,4]triazolo[4,3-a]pyrimidine-6-carboxylate* (**4b**), Yellow powder, yield: 796 mg (65%), Mp 141–143 °C; IR (KBr): ν_max_/cm^−1^ ν_C=O_: 1685, ν_C=N_: 1645. ^1^H NMR (400 MHz, CDCl_3_): δ = 1.26 (t, *J* = 6.4 Hz, 3H, CH_3_), 2.19 (s, 3H, CH_3_-C3), 2.53 (s, 3H, H10), 4.10–4,12 (m, 2H, O-CH_2_), 6.16 (s, 1H, H5), 7.29–7.32 (m, 5H), 7.48–7.52 (m, 2H), 8.19 (d, *J* = 7.4 Hz, 2H). ^13^C NMR (100 MHz, CDCl_3_): δ = 166.5 (C=O), 159.9 (C7), 148.4 (C3), 144.6 (C8a), 141.4, 137.5, 134.2, 129.0, 128.9, 128.5, 126.2, 120.0, 97.6 (C6), 59.6 (O-CH_2_), 57.0 (C5), 25.3 (C10), 14.4 (CH_3_), 11.0 (CH_3-_C3). HRMS (ESI): *m*/*z* [M + H]^+^ Calcd for C_22_H_22_ClN_4_O_2_: 409.1431; Found: 409.1424.

*Ethyl 5-(4-methoxyphenyl)-3,7-dimethyl-1-phenyl-1,5-dihydro-[1,2,4]triazolo[4,3-a]pyrimidine-6-carboxylate* (**4c**), Yellow powder, yield: 727 mg (60%), Mp 165–167 °C; IR (KBr): ν_max_/cm^−1^ ν_C=O_: 1692, ν_C=N_: 1642. ^1^H NMR (400 MHz, CDCl_3_): δ = 1.25 (t, *J* = 6.5 Hz, 3H, CH_3_ ), 2.20 (s, 3H, CH_3_-C3), 2.53 (s, 3H, H10), 3.79 (s, 3H, OCH_3_), 4.09–4.13 (m, 2H, O-CH_2_), 6.13 (s, 1H, H5), 6.83 (d, *J* = 7.6 Hz, 2H), 7.31 (d, J = 8.2 Hz, 3H), 7.46–7.49 (m, 2H), 8.19 (d, J = 7.3 Hz, 2H). ^13^C NMR (100 MHz, CDCl_3_): δ = 166.7 (C=O), 159.5 (C17), 159.3 (C7), 148.4 (C3), 144.8 (C8a), 137.6, 135.5, 129.0, 128.4, 126.0, 120.0, 113.9, 98.2 (C6), 59.5 (O-CH_2_), 57.0 (C5), 55.3 (OCH_3_), 25.2 (C10), 14.4 (CH_3_), 11.1 (CH-C3). HRMS (ESI): *m*/*z* [M + H]^+^ Calcd for C_23_H_25_N_4_O_3_: 405.1927; Found: 405.1920.

*Ethyl 3,7-dimethyl-1-phenyl-5-(p-tolyl)-1,5-dihydro-[1,2,4]triazolo[4,3-a]pyrimidine-6-carboxylate* (**4d**), Yellow powder, yield: 780 mg (67%), Mp 143–145 °C; IR (KBr): ν_max_/cm^−1^ ν_C=O_: 1688, ν_C=N_: 1643.5. ^1^H NMR (400 MHz, CDCl_3_): δ = 1.25 (t, *J* = 6.9 Hz, 3H, CH_3_), 2.20 (s, 3H, CH_3_-C3), 2.33 (s, 3H, CH_3_), 2.54 (s, 3H, H10), 4.109–4.13 (m, 2H, O-CH_2_), 6.14 (s, 1H, H_-_5), 7.12 (d, *J* = 7.4 Hz, 2H), 7.27 (d, *J* = 7.4 Hz, 3H), 7.47 (t, *J* = 7.8 Hz, 2H), 8.19 (d, *J* = 7.8 Hz, 2H). ^13^C NMR (100 MHz, CDCl_3_): δ = 166.6 (C=O), 159.5 (C7), 148.5 (C3), 144.8 (C8a), 140.1, 138.2, 137.7, 129.3, 129.0, 127.0, 126.0, 120.0, 98.1 (C6), 59.4 (O-CH_2_), 57.3 (C5), 25.2 (C10), 21.1 (CH_3_), 14.4 (CH_3_), 11.0 (CH_3_-C3). HRMS (ESI): *m*/*z* [M + H]^+^ Calcd for C_23_H_25_N_4_O_2_: 389.1978; Found: 389.1970.

*Ethyl 3,7-dimethyl-5-(4-nitrophenyl)-1-phenyl-1,5-dihydro-[1,2,4]triazolo[4,3-a]pyrimidine-6-carboxylate* (**4e**), Yellow powder, yield: 1005 mg (80%), Mp 164–166 °C; IR (KBr): ν_max_/cm^−1^ ν_C=O_: 1684, ν_C=N_: 1644. ^1^H NMR (400 MHz, CDCl_3_): δ = 1.27 (t, *J* = 7.1 Hz, 3H, CH_3_), 2.19 (s, 3H, CH_3_-C3), 2.53 (s, 3H, H10), 4.11–4.17 (m, 2H, O-CH_2_), 6.31 (s, 1H, H5), 7.32 (t, *J* = 7.4 Hz, 1H), 7.49 (t, *J* = 7.4 Hz, 2H), 7.58 (d, J = 8.7 Hz, 2H), 8.18–8.21 (m, 4H). ^13^C NMR (100 MHz, CDCl_3_): δ = 166.3 (C=O), 160.6 (C7), 149.2 (C3), 148.3, 147.8, 144.3 (C8a), 137.4, 129.1, 128.0, 126.4, 124.1, 120.1, 96.9 (C6), 59.8 (O-CH_2_), 56.9 (C5), 25.4 (C10), 14.4 (CH_3_), 11.0 (CH_3_-C3). HRMS (ESI): *m*/*z* [M + H]^+^ Calcd for C_22_H_22_N_5_O_4_: 420.1672; Found: 420.1664.

*Ethyl 5-(4-fluorophenyl)-3,7-dimethyl-1-phenyl-1,5-dihydro-[1,2,4]triazolo[4,3-a]pyrimidine-6-carboxylate* (**4f**), Yellow powder, yield: 882 mg (75%), Mp 181–183 °C; IR (KBr): ν_max_/cm^−1^ ν_C=O_: 1691, ν_C=N_: 1647. ^1^H NMR (400 MHz, CDCl_3_): δ = 1.25 (t, *J* = 7.0 Hz, 3H, CH_3_), 2.20 (s, 3H, CH_3_-C3), 2.53 (s, 3H, H10), 4.07–4.17 (m, 2H, O-CH_2_ ), 6.17 (s, 1H, H5 ), 7.01 (t, *J* = 8.4 Hz, 2H), 7.29 (d, *J* = 7.8 Hz, 1H), 7.37 (dd, *J* = 7.9, 5.5 Hz, 2H), 7.48 (t, *J* = 7.8 Hz, 2H), 8.19 (d, *J* = 7.8 Hz, 2H). ^13^C NMR (100 MHz, CDCl_3_): δ = 166.5 (C=O), 162.5 (C17), (d, *J* = 247.5 Hz), 159.7 (C7), 148.4 (C3), 144.6 (C8a), 139.0 (d, *J* = 3.2 Hz), 137.6, 129.0, 128.9 (d, *J* = 8.3 Hz), 126.2, 120.0, 115.6 (d, *J* = 21.7 Hz), 97.9 (C6), 59.5 (O-CH_2_), 56.9 (C5), 25.2 (C10), 14.4 (CH_3_), 11.0 (CH_3_-C3). HRMS (ESI): *m*/*z* [M + H]^+^ Calcd for C_22_H_22_FN_4_O_2_: 393.1727; Found: 393.1719.

*Ethyl 5-(2,4-dichlorophenyl)-3,7-dimethyl-1-phenyl-1,5-dihydro-[1,2,4]triazolo[4,3-a]pyrimidine-6-carboxylate* (**4g**), Yellow powder, yield: 1129 mg (85%), Mp 179–181 °C; IR (KBr): ν_max_/cm^−1^ ν_C=O_: 1687, ν_C=N_: 1641. ^1^H NMR (400 MHz, CDCl_3_): δ = 1.22 (t, *J* = 7.1 Hz, 3H, CH_3_), 2.27 (s, 3H, CH_3_-C3), 2.56 (s, 3H, H10), 4.11 (q, *J* = 7.1 Hz, 2H, O-CH_2_), 6.63 (s, 1H, H5 ), 7.23–7.25 (m, 1H), 7.30 (d, *J* = 7.7 Hz, 1H), 7.38 (d, *J* = 1.6 Hz, 1H), 7.45–7.50 (m, 3H), 8.16 (d, *J* = 8.0 Hz, 2H). ^13^C NMR (100 MHz, CDCl_3_): δ = 166.2 (C=O), 160.6 (C7), 148.2 (C3), 144.7 (C8a), 139.4, 137.4, 134.8, 131.9, 131.6, 129.2, 129.0, 128.6, 126.3, 120.1, 97.0 (C6), 59.6 (O-CH_2_), 53.6 (C5), 25.2 (C10), 14.5 (CH_3_), 11.4 (CH_3_-C3). HRMS (ESI): *m*/*z* [M + H]^+^ Calcd for C_22_H_21_Cl_2_N_4_O_2_: 443.1042; Found: 443.1034.

*Ethyl 3-ethyl-7-methyl-1,5-diphenyl-1,5-dihydro-[1,2,4]triazolo[4,3-a]pyrimidine-6-carboxylate* (**4h**), Yellow powder, yield: 920 mg (79%), Mp 169–171 °C; IR (KBr): ν_max_/cm^−1^ ν_C=O_: 1693, ν_C=N_: 1642. ^1^H NMR (400 MHz, CDCl_3_): δ = 1.21–1.27 (m, 6H, CH_3_-C3, CH_3_), 2.35–2.42 (m, 1H, CH_2_-C3), 2.53 (s, 3H, H10), 2.55–2.63 (m, 1H, CH_2_-C3), 4.07–4.16 (m, 2H, O-CH_2_), 6.20 (s, 1H, H5 ), 7.26–7.33 (m, 4H), 7.38–7.40 (m, 2H), 7.48 (t, *J* = 7.9 Hz, 2H), 8.22 (d, *J* = 7.9 Hz, 2H). ^13^C NMR (100 MHz, CDCl_3_): δ = 166.6 (C=O), 159.7 (C7), 148.9 (C3), 148.6 (C8a), 143.1, 137.7, 129.0, 128.7, 128.3, 127.0, 126.0, 120.1, 97.9 (C6), 59.5 (O-CH_2_), 57.4 (C5), 25.2 (C10), 18.7 (CH_2_-C3), 14.4 (CH_3_), 9.9 (CH_3_-C3). HRMS (ESI): *m*/*z* [M + H]^+^ Calcd for C_23_H_25_N_4_O_2_: 389.1978; Found: 389.1970.

*Ethyl 5-(4-chlorophenyl)-3-ethyl-7-methyl-1-phenyl-1,5-dihydro-[1,2,4]triazolo[4,3-a]pyrimidine-6-carboxylate* (**4i**), Yellow powder, yield: 861 mg (68%), Mp 148–151 °C; IR (KBr): ν_max_/cm^−1^ ν_C=O_: 1691, ν_C=N_: 1640. ^1^H NMR (400 MHz, CDCl_3_): δ = 1.23–1.28 (m, 6H, CH_3_-C3, CH_3_), 2.34–2.43 (m, 1H, CH_2_-C3), 2.52 (s, 3H, H10), 2.54–2.62 (m, 1H, CH_2_-C3), 4.08–4.17 (m, 2H, O-CH_2_), 6.18 (s, 1H, H5), 7.28–7.34 (m, 5H), 7.48 (t, *J* = 7.9 Hz, 2H), 8.21 (d, *J* = 7.9 Hz, 2H). ^13^C NMR (100 MHz, CDCl_3_): δ = 166.5 (C=O), 159.9 (C7), 148.7 (C3), 148.5 (C8a), 141.5, 137.6, 134.2, 129.0, 128.9, 128.4, 126.2, 120.1, 97.6 (C6), 59.6 (O-CH_2_), 56.8 (C5), 25.3 (C10), 18.7 (CH_2_-C3), 14.4 (CH_3_), 9.8 (CH_3_-C3). HRMS (ESI): *m*/*z* [M + H]^+^ Calcd for C_23_H_24_ClN_4_O_2_: 423.1588; Found: 423.1582.

*Ethyl 3-ethyl-5-(4-methoxyphenyl)-7-methyl-1-phenyl-1,5-dihydro-[1,2,4]triazolo[4,3-a]pyrimidine-6-carboxylate* (**4j**), Yellow powder, yield: 727 mg (58%), Mp 138–140 °C; IR (KBr): ν_max_/cm^−1^ ν_C=O_: 1692, ν_C=N_: 1648. ^1^H NMR (400 MHz, CDCl_3_): δ = 1.22–1.27 (m, 6H, CH_3_-C3, CH_3_), 2.39–2.45 (m, 1H, CH_2_-C3), 2.53 (s, 3H, H10), 2.55–2.62 (m, 1H, CH_2_-C3), 3.79 (s, 3H, OCH_3_), 4.10–4.14 (m, 2H, O-CH_2_), 6.15 (s, 1H, H5), 6.83 (d, *J* = 8.1 Hz, 2H), 7.26–7.31 (m, 3H), 7.48 (t, *J* = 7.9 Hz, 2H), 8.22 (d, *J* = 7.9 Hz, 2H). ^13^C NMR (100 MHz, CDCl_3_): δ = 166.7 (C=O), 159.4 (C17), 159.3 (C7), 148.9 (C3), 148.5 (C8a), 137.8, 135.6, 129.0, 128.3, 126.0, 120.0, 113.9, 98.2 (C6), 59.4 (O-CH_2_), 56.8 (C3), 55.3 (OCH_3_), 25.2 (C10), 18.7 (CH_2_-C3), 14.4 (CH_3_), 9.9 (CH_3_-C3). HRMS (ESI): *m*/*z* [M + H]^+^ Calcd for C_24_H_27_N_4_O_3_: 419.2083; Found: 419.2075.

*Ethyl 3-ethyl-7-methyl-1-phenyl-5-(p-tolyl)-1,5-dihydro-[1,2,4]triazolo[4,3-a]pyrimidine-6-carboxylate* (**4k**), Yellow powder, yield: 748 mg (62%), Mp 137–139 °C; IR (KBr): ν_max_/cm^−1^ ν_C=O_: 1690, ν_C=N_: 1645. ^1^H NMR (400 MHz, CDCl_3_): δ = 1.22–1.28 (m, 6H, CH_3_-C3, CH_3_), 2.32 (s, 3H, CH_3_), 2.40–2.46 (m, 1H, CH_2_-C3), 2.53 (s, 3H, H10), 2.55–2.61 (m, 1H, CH_2_-C3), 4.05–4.17 (m, 2H, O-CH_2_), 6.16 (s, 1H, H5), 7.11 (d, *J* = 7.4 Hz, 2H), 7.26–7.28 (m, 3H), 7.48 (t, *J* = 7.9 Hz, 2H), 8.22 (d, *J* = 7.9 Hz, 2H). ^13^C NMR (100 MHz, CDCl_3_): δ = 166.7 (C=O), 159.5 (C7), 148.9 (C3), 148.6 (C8a), 140.2, 138.1, 137.8, 129.3, 129.0, 126.9, 126.0, 120.1, 98.1 (C6), 59.4 (O-CH_2_), 57.1 (C5), 25.2 (C10), 21.1 (CH_3_), 18.7 (CH_2_-C3), 14.5 (CH_3_), 9.9 (CH_3_-C3). HRMS (ESI): *m*/*z* [M + H]^+^ Calcd for C_24_H_27_N_4_O_2_: 403.2134; Found: 403.2127.

*Ethyl 3-ethyl-7-methyl-5-(4-nitrophenyl)-1-phenyl-1,5-dihydro-[1,2,4]triazolo[4,3-a]pyrimidine-6-carboxylate* (**4l**), Yellow powder, yield: 1104 mg (85%), Mp 161–163 °C; IR (KBr): ν_max_/cm^−1^ ν_C=O_: 1691, ν_C=N_: 1643. ^1^H NMR (400 MHz, CDCl_3_): δ = 1.24–1.29 (m, 6H, CH_3_-C3, CH_3_), 2.30–2.39 (m, 1H, CH_2_-C3), 2.52 (s, 3H, H10), 2.55–2.63 (m, 1H, CH_2_-C3), 4.08–4.18 (m, 2H, O-CH_2_), 6.32 (s, 1H, H5), 7.31–7.33 (m, 1H), 7.50 (t, *J* = 7.9 Hz, 2H), 7.57 (d, *J* = 8.6 Hz, 2H), 8.18–8.22 (m, 4H). ^13^C NMR (100 MHz, CDCl_3_): δ = 166.4 (C=O), 160.6 (C7), 149.3 (C3), 148.4 (C8a), 148.4, 147.7, 137.5, 129.1, 127.9, 126.4, 124.1, 120.1, 96.9 (C6), 59.8 (O-CH_2_), 56.7 (C5), 25.4 (C10), 18.7 (CH_2_-C3), 14.4 (CH_3_), 9.8 (CH_3_-C3). HRMS (ESI): *m*/*z* [M + H]^+^ Calcd for C_23_H_24_N_5_O_4_: 434.1828; Found: 434.1820.

*Ethyl 3-ethyl-5-(4-fluorophenyl)-7-methyl-1-phenyl-1,5-dihydro-[1,2,4]triazolo[4,3-a]pyrimidine-6-carboxylate* (**4m**), (CH_3_-C7)^1^H NMR (400 MHz, CDCl_3_): δ = 1.23–1.27 (m, 6H, CH_3_-C3, CH_3_), 2.36–2.44 (m, 1H, CH_2_-C3), 2.53 (s, 3H, H10), 2.55–2.61 (m, 1H, CH_2_-C3), 4.07–4.17 (m, 2H, O-CH_2_), 6.19 (s, 1H, H5), 7.00 (t, *J* = 8.5 Hz, 2H), 7.30–7.33 (m, 1H), 7,36 (dd, *J* = 8.5, 5.3 Hz, 2H), 7.48 (t, *J* = 7.9 Hz, 2H), 8.21 (d, *J* = 7.9 Hz, 2H). ^13^C NMR (100 MHz, CDCl_3_): δ = 166.6 (C=O), 162.5 (C17) (d, *J* = 247.2 Hz), 159.7 (C7), 148.7 (C3), 148.5 (C8a), 139.1 (d, J = 3.2 Hz), 137.7, 129.0, 128.8 (d, *J* = 8.4 Hz), 126.1, 120.1, 115.6 (d, J = 21.7 Hz), 97.9 (C6), 59.5 (O-CH_2_), 56.7 (C5), 25.2 (C10), 18.7 (CH_2_-C3), 14.4 (CH_3_), 9.9 (CH_3_-C3). HRMS (ESI): *m*/*z* [M + H]^+^ Calcd for C_23_H_24_FN_4_O_2_: 407.1883; Found: 407.1877.

*Ethyl 5-(2,4-dichlorophenyl)-3-ethyl-7-methyl-1-phenyl-1,5-dihydro-[1,2,4]triazolo[4,3-a]pyrimidine-6-carboxylate* (**4n**), Yellow powder, yield: 1094 mg (80%), Mp 179–181 °C; IR (KBr): ν_max_/cm^−1^ ν_C=O_: 1685, ν_C=N_: 1642. ^1^H NMR (400 MHz, CDCl_3_): δ = 1.21–1.29 (m, 6H, CH_3_-C3, CH_3_), 2.38–2.48 (m, 1H, CH_2_-C3), 2.56 (s, 3H, H10), 2.66–2.75 (m, 1H, CH_2_-C3), 4.09–4.14 (m, 2H, O-CH_2_), 6.63 (s, 1H, H5), 7.23 (dd, *J* = 8.4, 2.0 Hz, 1H), 7.28 (t, *J* = 7.7 Hz, 1H), 7.38 (d, *J* = 2.0 Hz, 1H), 7.45–7.50 (m, 3H), 8.18 (d, *J* = 7.7 Hz, 2H). ^13^C NMR (100 MHz, CDCl_3_): δ = 166.2 (C=O), 160.6 (C7), 148.9 (C3), 148.3 (C8a), 139.5, 137.5, 134.7, 131.5, 129.1, 129.0, 128.5, 126.2, 120.2, 97.1 (C6), 59.5 (O-CH_2_), 53.5 (C5), 25.2 (C10), 18.8 (CH_2_-C3), 14.5 (CH_3_), 10.2 (CH_3_-C3). HRMS (ESI): *m*/*z* [M + H]^+^ Calcd for C_23_H_23_Cl_2_N_4_O_2_: 457.1198; Found: 457.1193.

### 3.2. Pharmacology

We adopted the same method as Msalbi et al. [42]. Compounds were solubilized in DMSO and serially diluted with cell culture media just before use.

For MCF-7 and T47D, the cell lines are obtained from the Institut Pasteur in Tunis (Tunisia). For line MDA-MB-231, it is obtained from our colleague Mohamed Abdelkarim, Faculty of Medicine of Tunis. This line was the subject of two publications by our colleague.

Human breast cancer cell lines MDA-MB-231 (ER-, PR-, HER2-) and MCF7 (ER+, PR+, HER2-) were cultured in DMEM medium supplemented with 10% fetal bovine serum, 50 IU/mL penicillin and 50 mg/mL streptomycin and maintained in an incubator at 37 °C in a humidified atmosphere at 5% CO_2_. The MTT test was performed to determine the effect of our chemical compounds on breast cancer cell lines MDA-MB-231 and MCF7 compared to Cisplatin. A 96-well microtiter plate containing 0.1 mL DMEM/well was seeded with 8.103 of each cell line. After 24 h of culture, the cells were treated with these compounds and Cisplatin for 48 h with different concentrations ranging from 0 to 100 µM. After exposure, the medium was removed and MTT solution was added (5 mg/mL) to each well containing 100 µL of DMEM. After 4 h, a formazan precipitate was dissolved in 100 µL/well 10% SDS and recorded at 570 nm. The IC_50_ value was defined as cell viability ratio (%) = (OD treated/OD untreated) × 100. All the experiments were carried out in triplicate with six measurements for each concentration tested.

## 4. Conclusions

In conclusion, we have developed a simple and efficient strategy for preparation of [1,2,4]triazolo[4,3-*a*]pyrimidine derivatives through a one-pot three-component reaction from easily available 5-amino-1-phenyl-1*H*-1,2,4-triazoles, aromatic aldehydes and ethyl acetoacetate using 10 mol% APTS as a catalyst. All synthesized products are new and have been isolated with good yields. The regioselectivity of this developed condensation was confirmed by 2D NMR analysis and X-ray crystallographic analyses. Fourteen of the newly synthesized compounds were screened for their biological activities and found to be more active against the tumor cell lines MDA-MB-321 and MCF-7 compared to Cisplatin. The results showed that among this series, compounds **4c** and **4j** exhibited the best antitumor activity against breast cancer with respective IC_50_ values of 17.83 μM, and 19.73 μM. These will be taken into account in further study.

## Figures and Tables

**Table 1:** Optimization of reaction conditions for substituted [1,2,4]triazolo[4,3-*a*]pyrimidines **4a**.

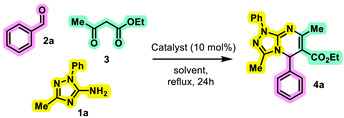			
Entry	Solvent	Catalyst	Yield (%) ^a^
1	H2O	APTS	Trace
**2**	**Ethanol**	**APTS**	**75**
3	Ethanol	HCl	45
4	Ethanol	Acetic acid	10
5	Ethanol	Piperidine	Trace
6	CH3CN	APTS	50
7	Fusion state	-	10

^a^ Isolated yield.

**Table 2:** IC_50_ of compounds **4a**–**n** and Cisplatin against MDA-MB-231 and MCF7 after 48 h of treatment activities.

Compound	IC_50_ (μM)		Compound	IC_50_ (μM)	
	MDA-MB-231	MCF-7		MDA-MB-231	MCF-7
**4a**	41.30 ± 1.8	43.86 ± 3.1	**4h**	>100	>100
**4b**	38.31 ± 2	36.28 ± 3.3	**4i**	43.88 ± 1	>100
**4c**	17.83 ± 2	20.33 ± 1.7	**4j**	23.97 ± 2.7	19.73 ± 1.5
**4d**	44.63 ± 0.3	34.20 ± 1	**4k**	53.45 ± 4.9	48.45 ± 1.1
**4e**	62.26 ± 1.7	>100	**4l**	>100	>100
**4f**	43.79 ± 1.8	32.09 ± 2.8	**4m**	84.43 ± 3.8	>100
**4g**	39.57 ± 0.3	51.26 ± 0.7	**4n**	58.34 ± 3.4	46.34 ± 2
Cisplatin	38.6 ± 0.8	83.1 ± 1.3	Cisplatin	38.6 ± 0.8	83.1 ± 1.3

## Data Availability

The data set presented in this study is available in this article.

## Supplementary Materials

The following supporting information can be downloaded at: https://www.mdpi.com/article/10.3390/molecules28093917/s1, Figures S1–S26: 1H and 13C NMR spectra of **4a–n**; Figure S27: 2D NMR HMBC sequence of compound **4f**; Figure S28: FT-IR spectrum of **4n**; Copies of HRMS report (in French).

## Sample Availability

Samples of all compounds are available from the authors.
