# Supplementary material for: Three Component One-Pot Synthesis and Antiproliferative Activity of New [1,2,4]Triazolo[4,3-a]pyrimidines
Source: Molecules. 2023 May 5;28(9):3917. doi: 10.3390/molecules28093917 (PMC10180348; doi:10.3390/molecules28093917)
Supplement: Supplementary file 1 [file molecules-28-03917-s001.zip › molecules-2318165-supplementary.pdf]

Supplementary Materials

Three Component One-Pot Synthesis and Antiproliferative Activity of New [1,2,4]Triazolo[4,3-*a*]Pyrimidines.

Manel Ben Hassen [1], Dhouha Msalbi [2], Badr Jismy [3], Fares Elghali [2], Sami Aifa [2], Hassan Allouchi [4], Mohamed Abarbri [3,*], Fakher Chabchoub [1]

[1] Laboratory of Applied Chemistry: Heterocycles, Lipids, and Polymers, Faculty of Sciences of Sfax, University of Sfax, BP 802, Sfax 3000, Tunisia.

[2] Laboratory of Molecular and Cellular Screening Processes, Centre of Biotechnology of Sfax, Sidi Mansour, Road Km 6, BP 1177, Sfax 3018, Tunisia.

[3] Laboratory of Physico-Chemistry of Materials and Electrolytes for Energy (PCM2E), EA 6299. University of Tours, Faculty of Science and Technology, 37200 Tours, France.

[4] Faculty of Pharmacy, University of Tours, EA 7502 SIMBA, 31 Avenue Monge, 37200 Tours, France.

* Correspondence: mohamed.abarbri@univ-tours.fr

Table of Contents

1. Figures S1-S26: ^{1}H and ^{13}C NMR spectra of **4a-n** S3-S16

2. Figure S27: 2D NMR HMBC sequence of compound **4f** S17

3. Figure S28: FT-IR spectrum of **4n** S17

4. Copies of HRMS report (in French) S18-S27

Ethyl 3,7-dimethyl-1,5-diphenyl-1,5-dihydro-[1,2,4]triazolo[4,3-*a*]pyrimidine-6-carboxylate 4a

Figure S1: ^{1}H NMR (400 MHz, $CDCl_3$) spectra of **4a**.

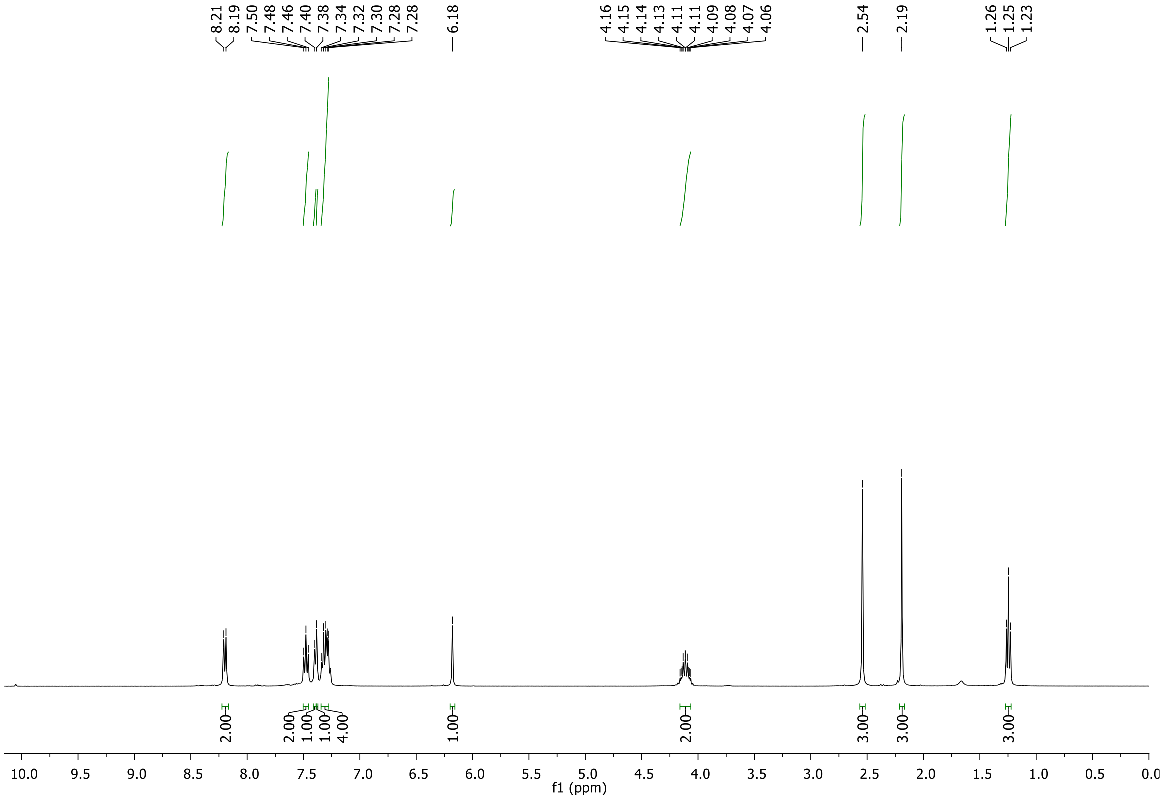

Figure S2: ^{13}C NMR (100 MHz, $CDCl_3$) spectra of **4a**.

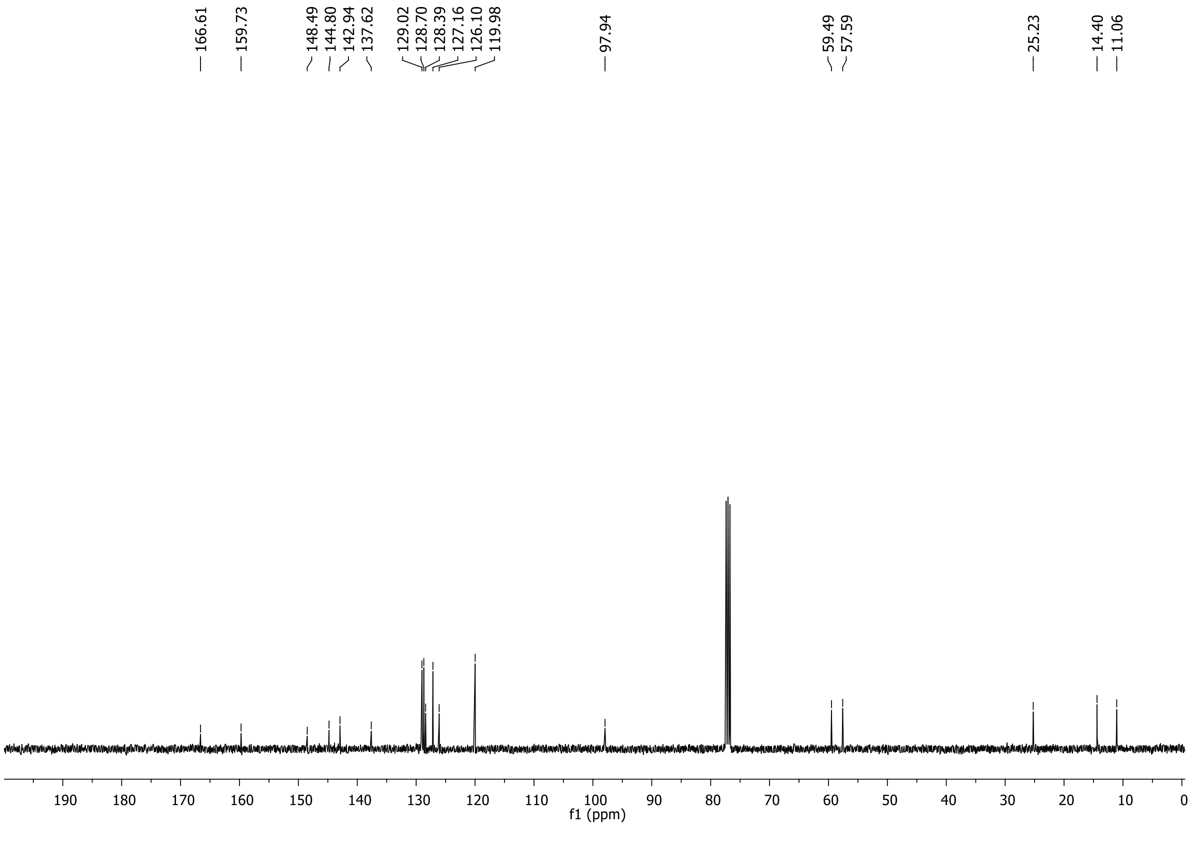

Ethyl 5-(4-chlorophenyl)-3,7-dimethyl-1-phenyl-1,5-dihydro-[1,2,4]triazolo[4,3-*a*]pyrimidine-6-carboxylate 4b

Figure S3: ^{1}H NMR (400 MHz, $CDCl_3$) spectra of **4b**.

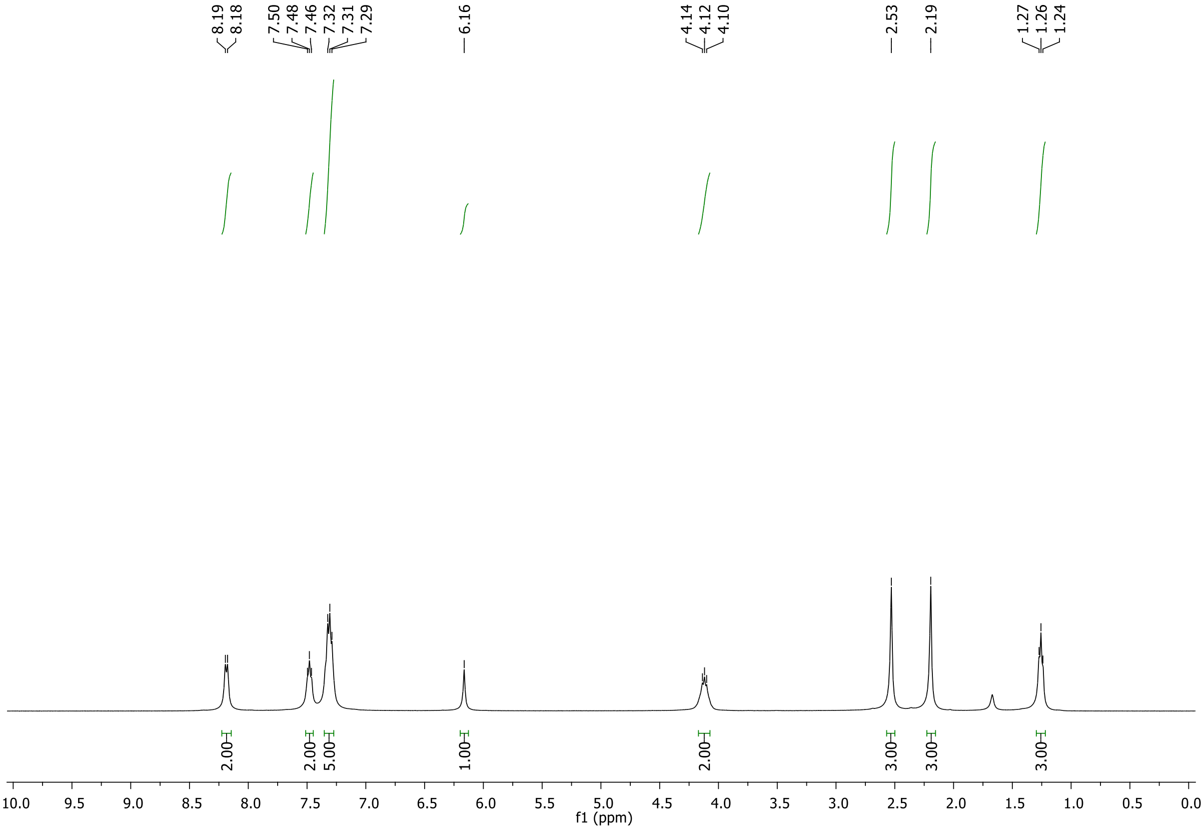

Figure S4: ^{13}C NMR (100 MHz, $CDCl_3$) spectra of **4b**.

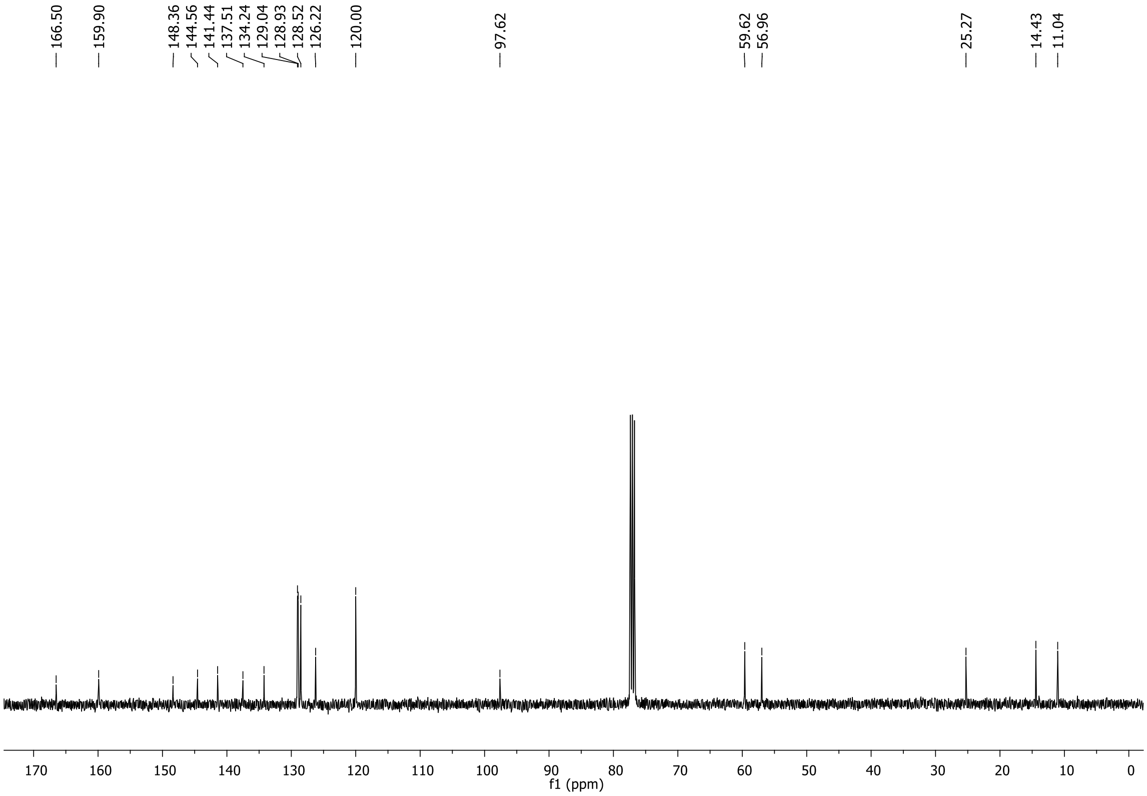

Ethyl 5-(4-methoxyphenyl)-3,7-dimethyl-1-phenyl-1,5-dihydro-[1,2,4]triazolo[4,3-a]pyrimidine-6-carboxylate 4c

Figure S5: ^{1}H NMR (400 MHz, CDCl$_3$) spectra of **4c**.

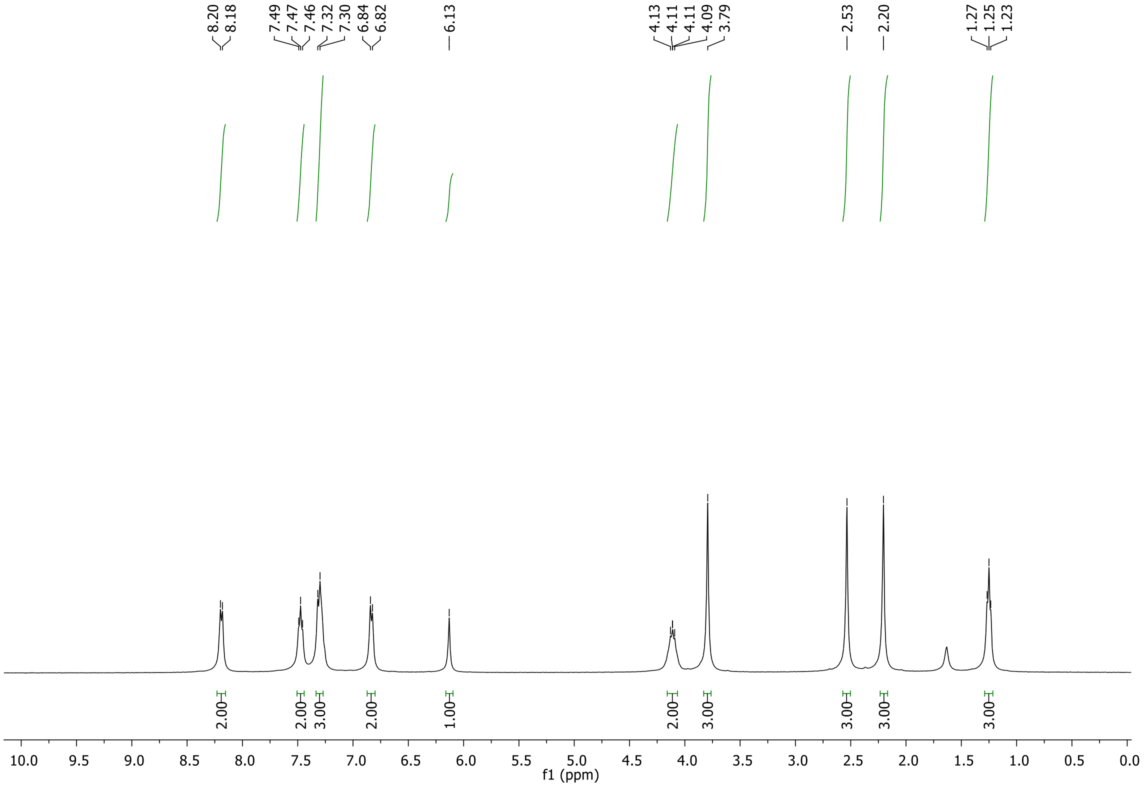

Figure S6: ^{13}C NMR (100 MHz, CDCl$_3$) spectra of **4c**.

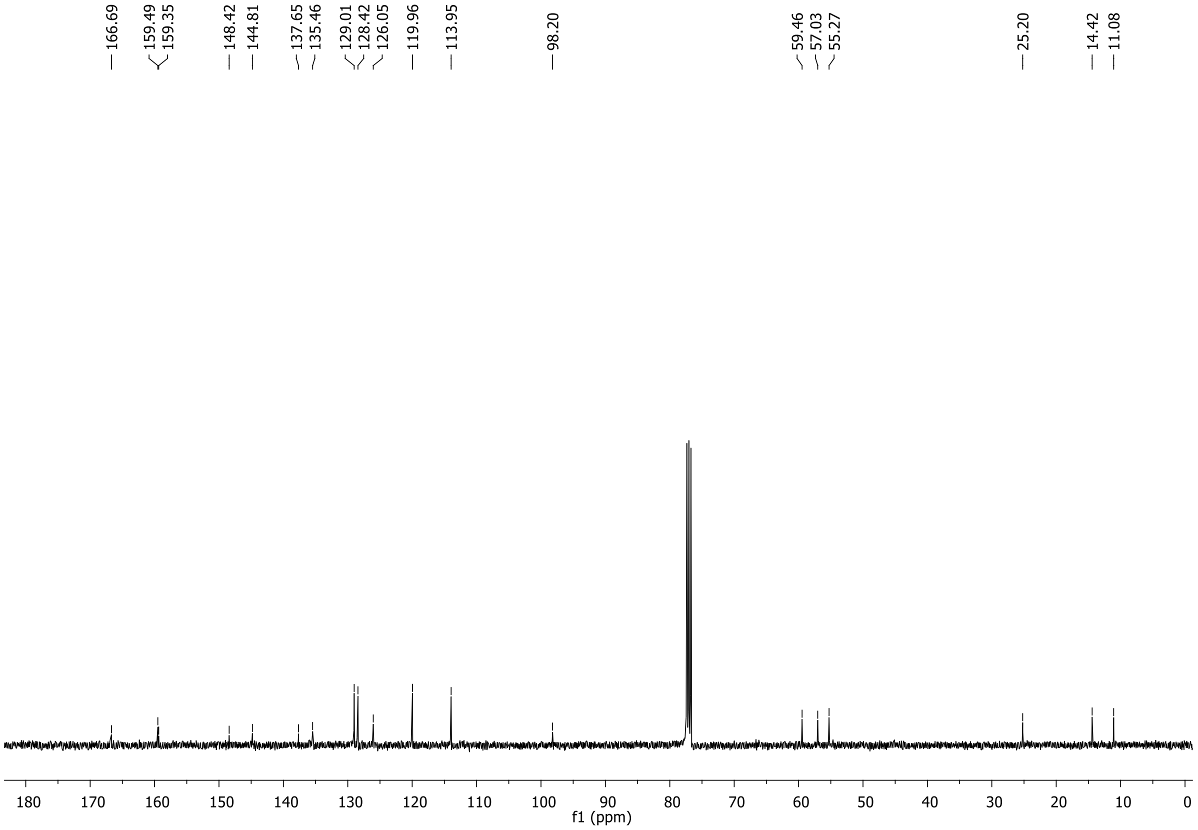

Ethyl 3,7-dimethyl-1-phenyl-5-(*p*-tolyl)-1,5-dihydro-[1,2,4]triazolo[4,3-*a*]pyrimidine-6-carboxylate 4d

Figure S7: ^{1}H NMR (400 MHz, $CDCl_3$) spectra of **4d**.

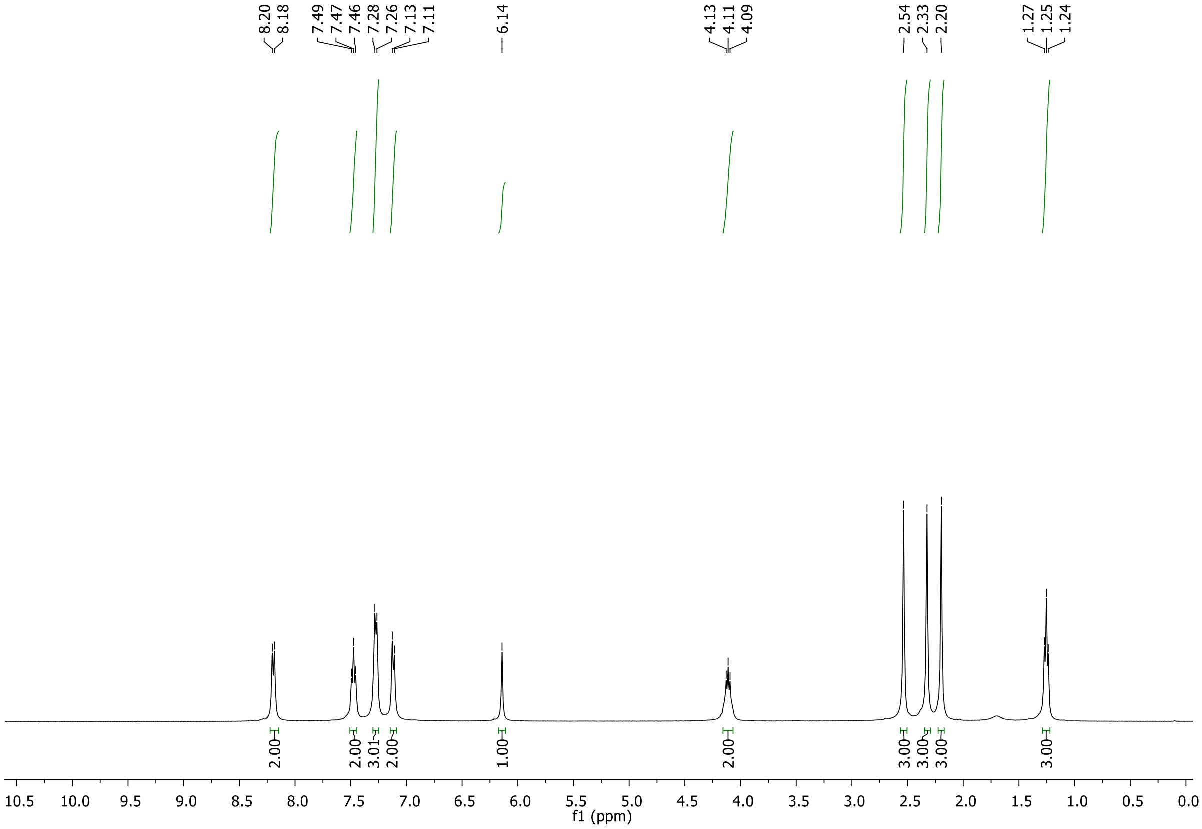

Figure S8: ^{13}C NMR (100 MHz, $CDCl_3$) spectra of **4d**.

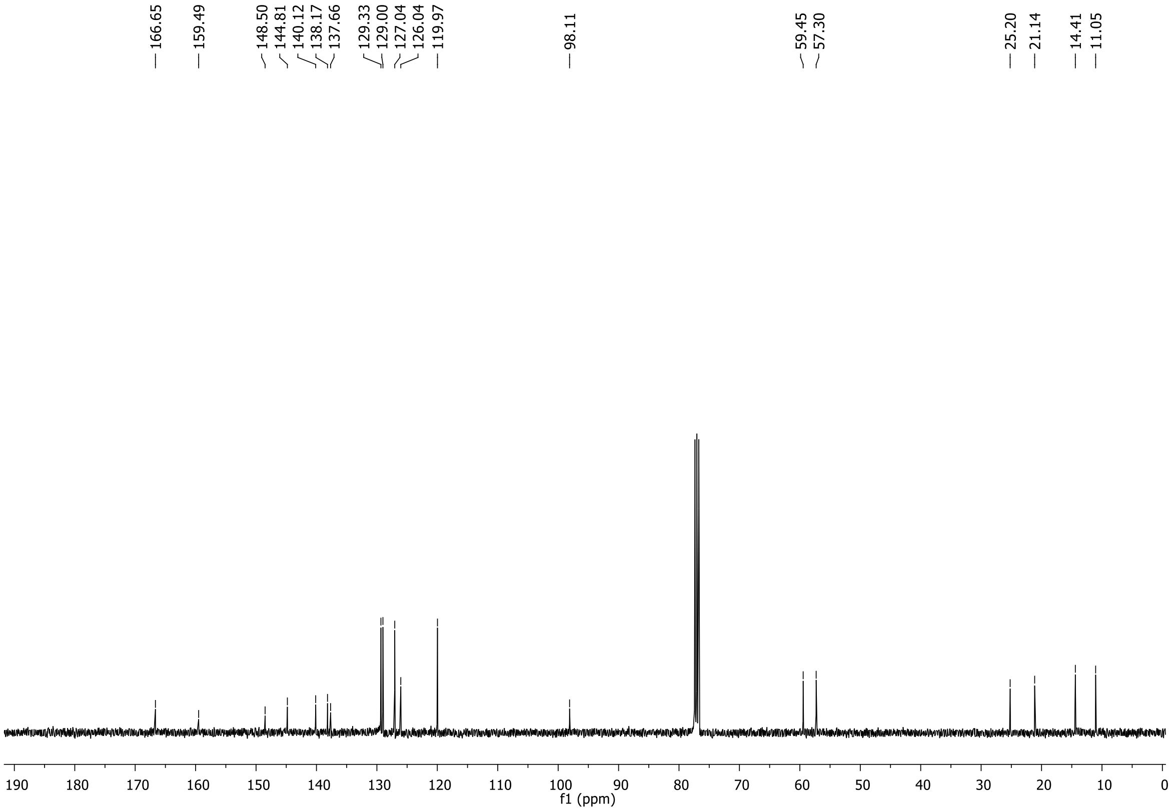

Ethyl 3,7-dimethyl-5-(4-nitrophenyl)-1-phenyl-1,5-dihydro-[1,2,4]triazolo[4,3-a]pyrimidine-6-carboxylate 4e

Figure S7: ^{1}H NMR (400 MHz, $CDCl_3$) spectra of **4e**.

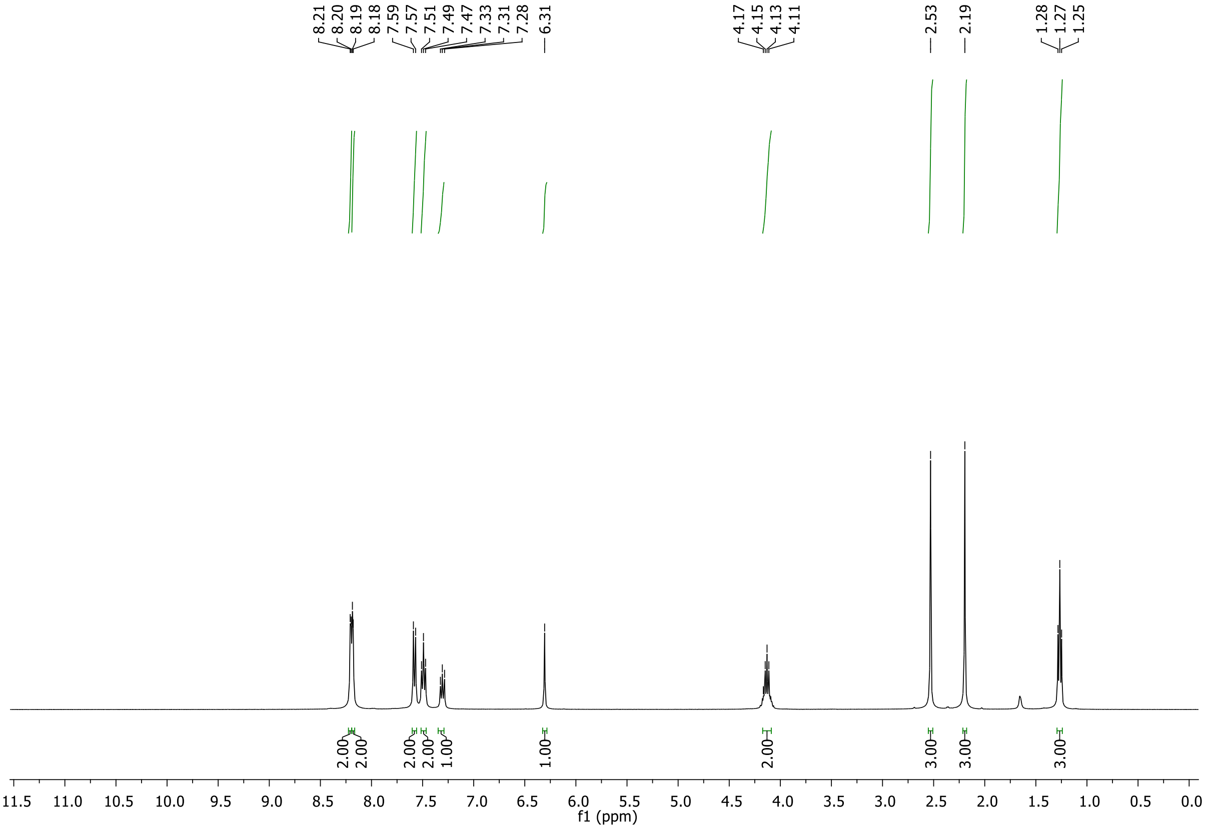

Figure S8: ^{13}C NMR (100 MHz, $CDCl_3$) spectra of **4e**.

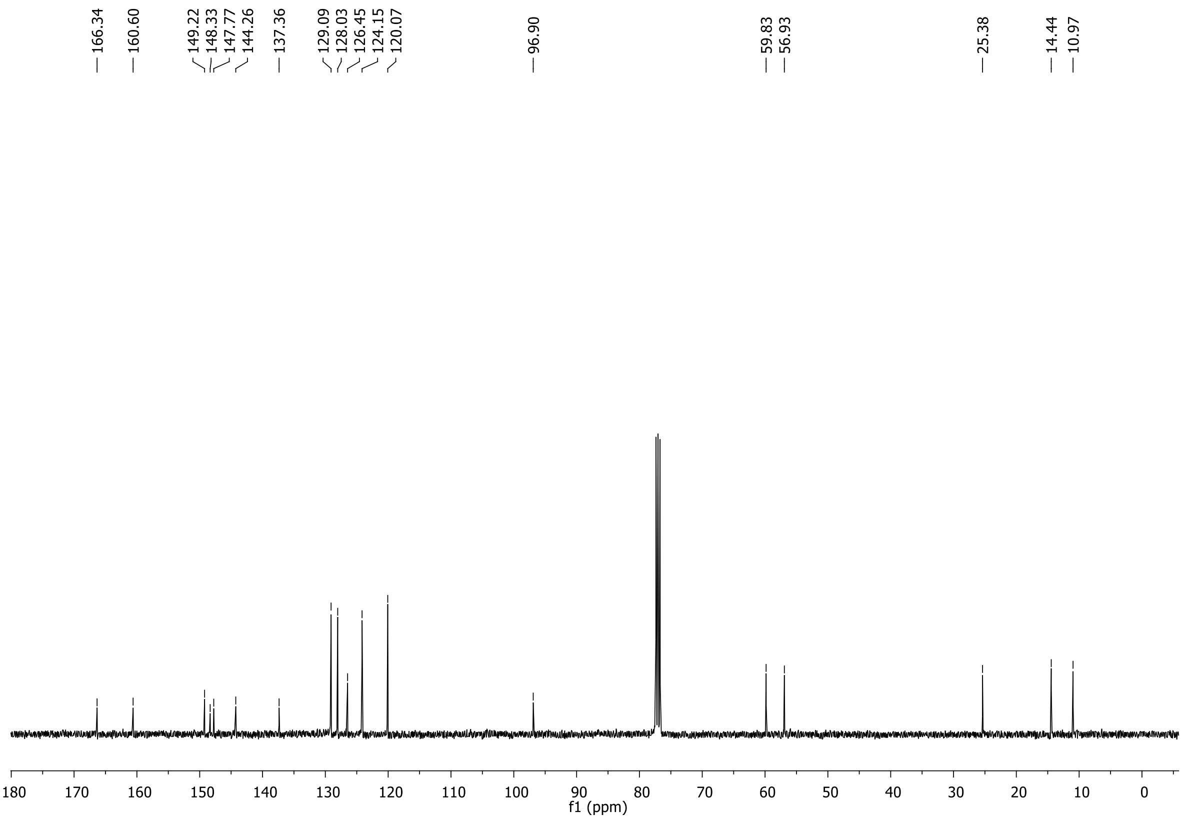

Ethyl 5-(4-fluorophenyl)-3,7-dimethyl-1-phenyl-1,5-dihydro-[1,2,4]triazolo[4,3-*a*]pyrimidine-6-carboxylate 4f

Figure S9: ^{1}H NMR (400 MHz, $CDCl_3$) spectra of **4f**.

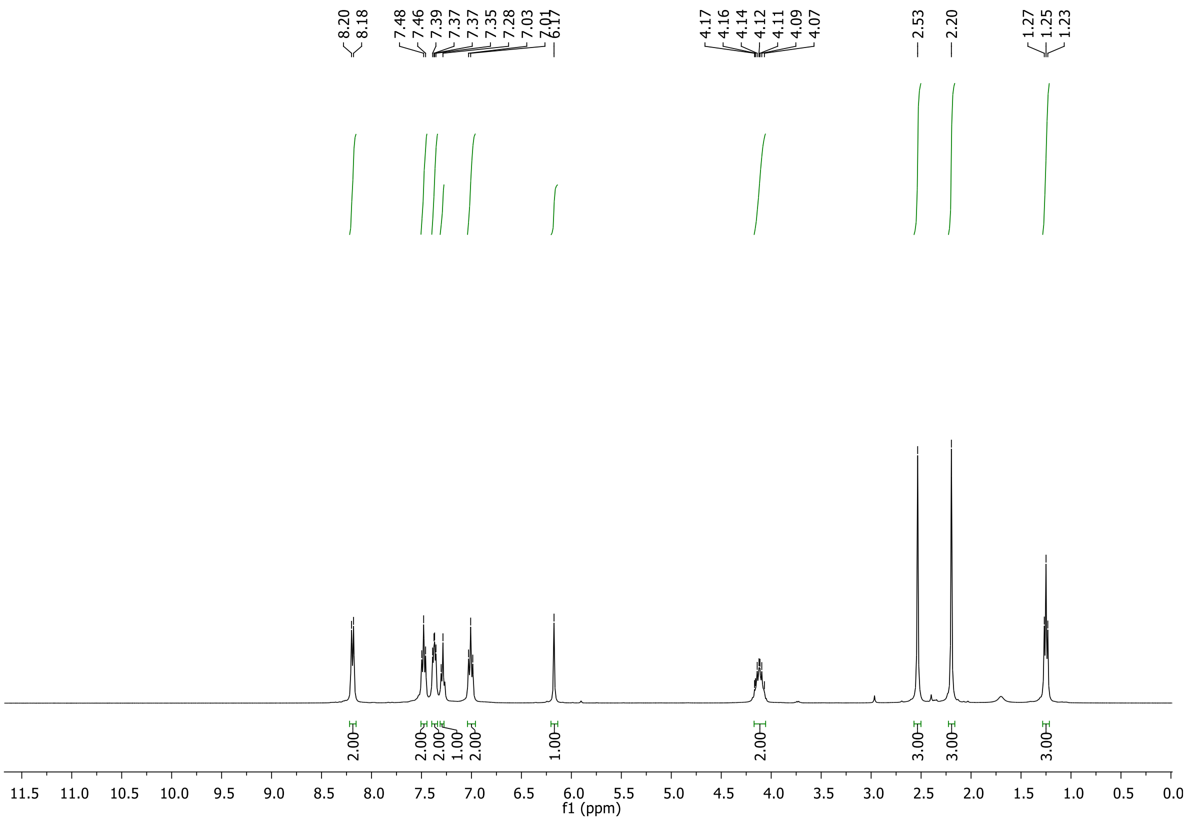

Figure S10: ^{13}C NMR (100 MHz, $CDCl_3$) spectra of **4f**.

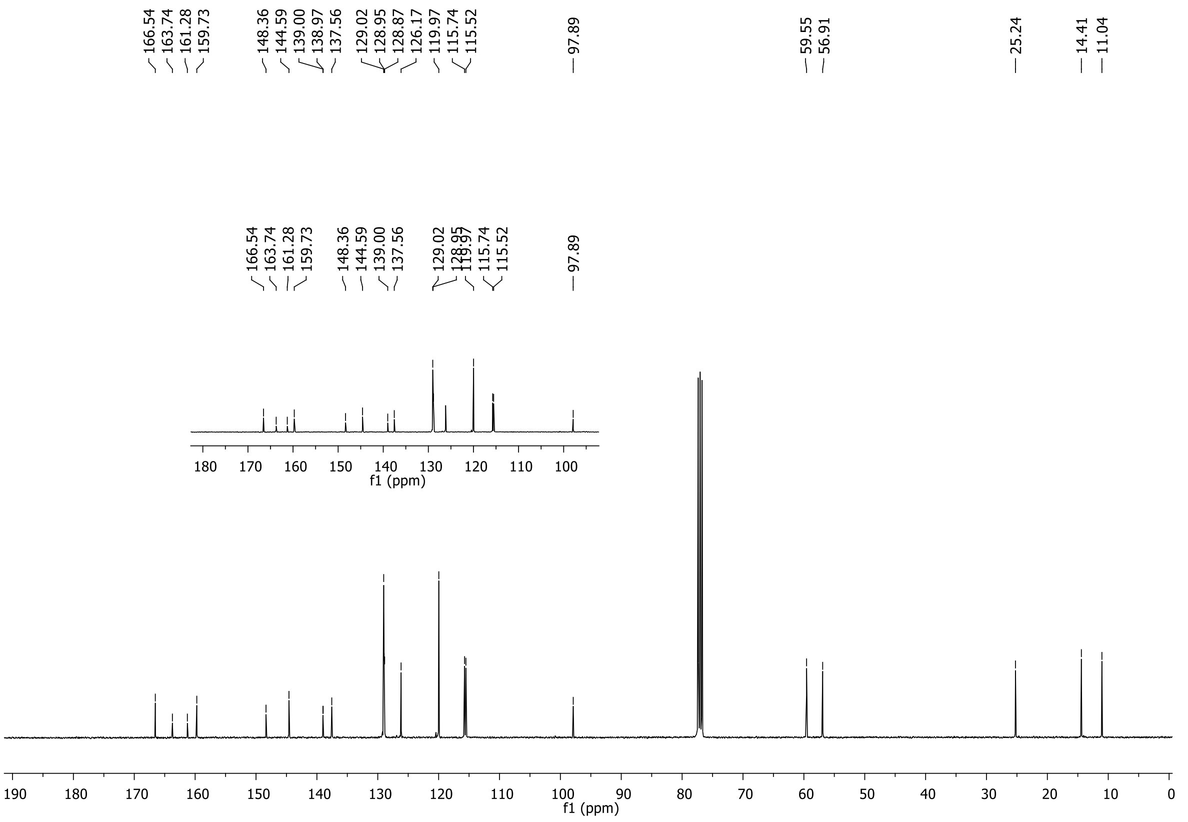

Ethyl 5-(2,4-dichlorophenyl)-3,7-dimethyl-1-phenyl-1,5-dihydro-[1,2,4]triazolo[4,3-*a*]pyrimidine-6-carboxylate 4g

Figure S11: ^{1}H NMR (400 MHz, $CDCl_3$) spectra of **4g**.

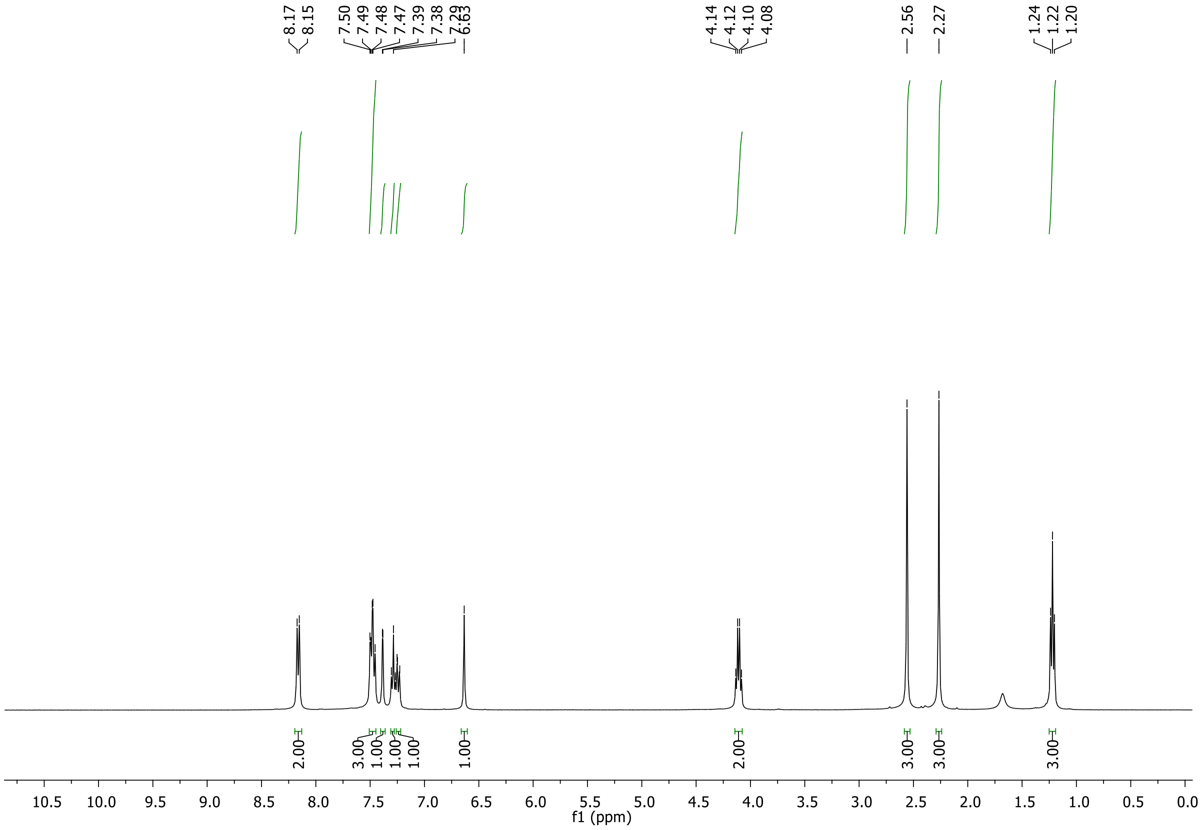

Figure S12: ^{13}C NMR (100 MHz, $CDCl_3$) spectra of **4g**.

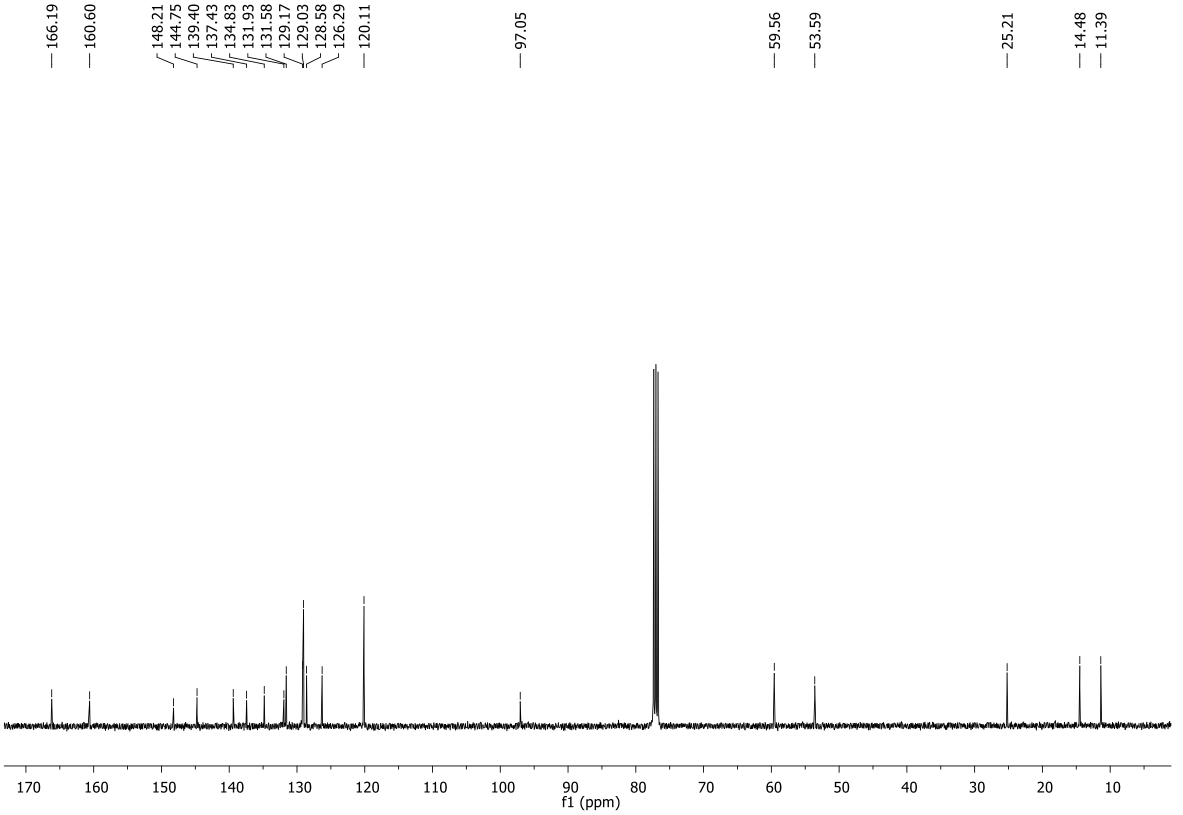

Ethyl 3-ethyl-7-methyl-1,5-diphenyl-1,5-dihydro-[1,2,4]triazolo[4,3-*a*]pyrimidine-6-carboxylate 4h

Figure S13: ^{1}H NMR (400 MHz, $CDCl_3$) spectra of **4h**.

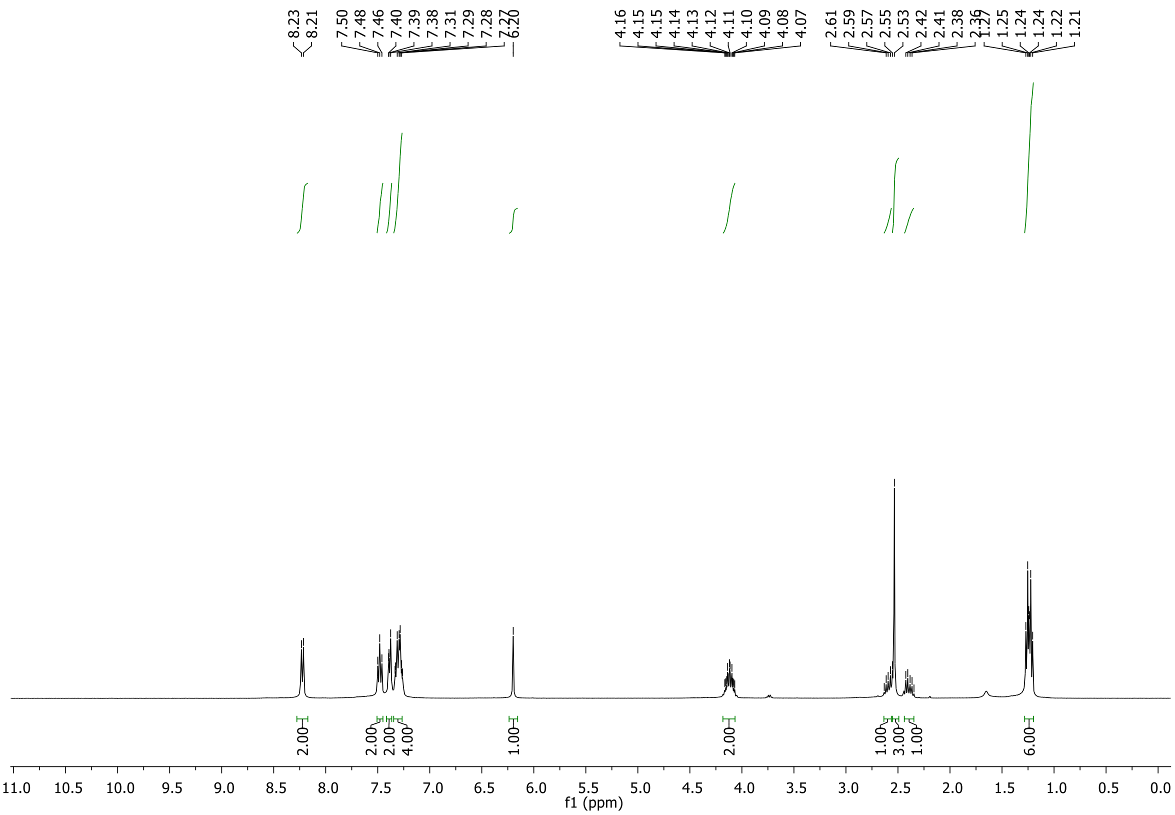

Figure S14: ^{13}C NMR (100 MHz, $CDCl_3$) spectra of **4h**.

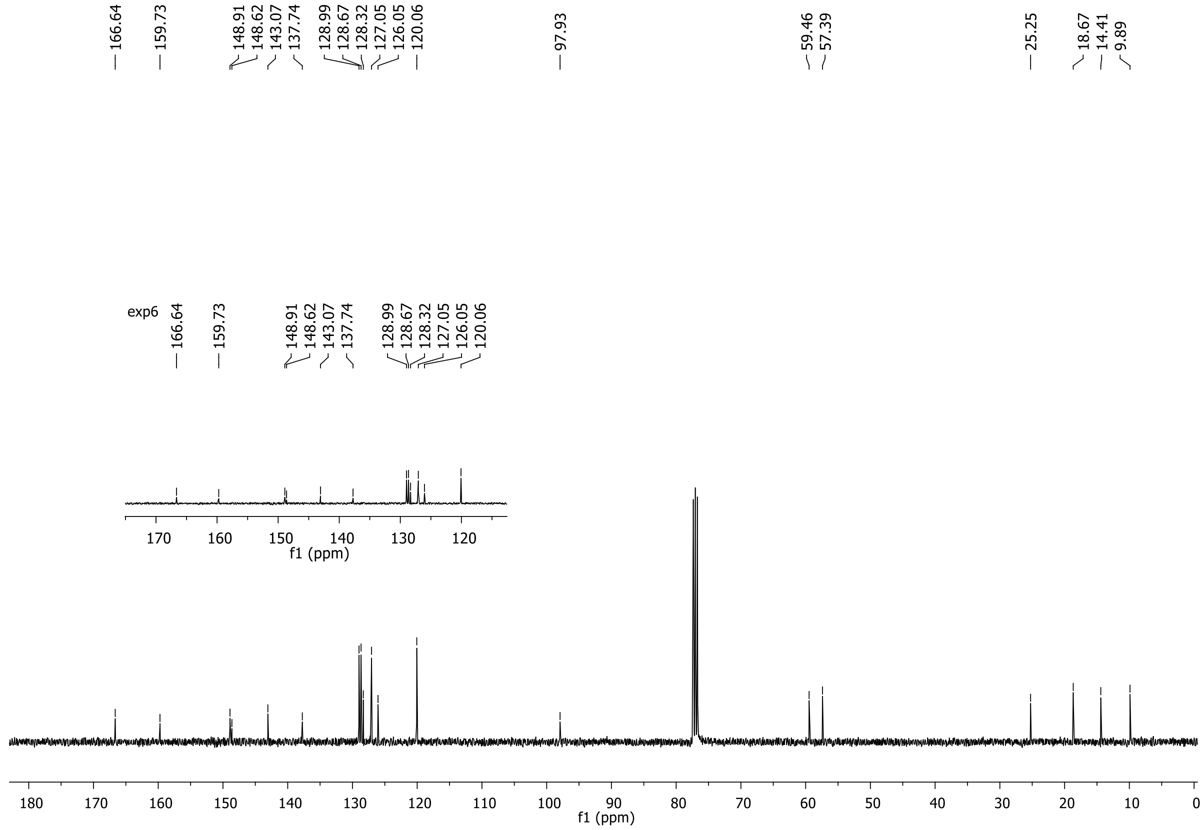

Ethyl 5-(4-chlorophenyl)-3-ethyl-7-methyl-1-phenyl-1,5-dihydro-[1,2,4]triazolo[4,3-*a*]pyrimidine-6-carboxylate 4i

Figure S15: ^{1}H NMR (400 MHz, $CDCl_3$) of spectra **4i**.

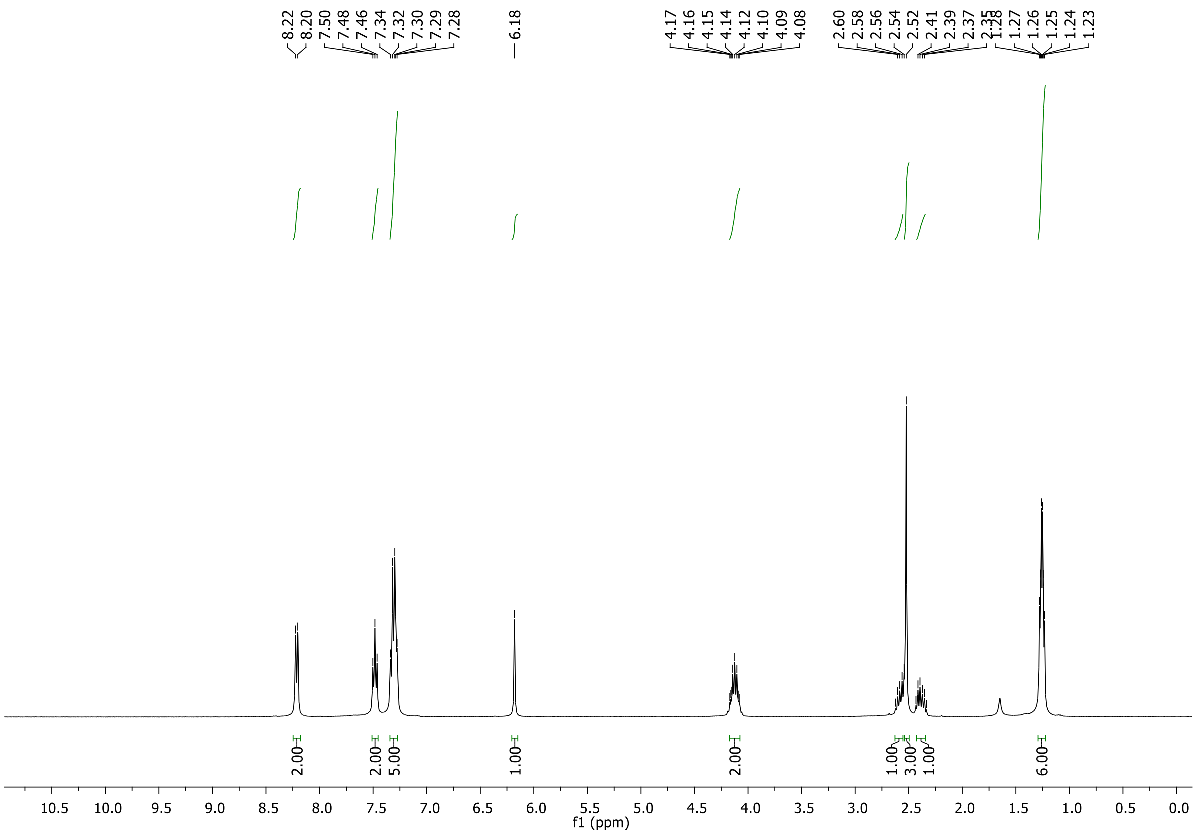

Figure S16: ^{13}C NMR (100 MHz, $CDCl_3$) spectra of **4i**.

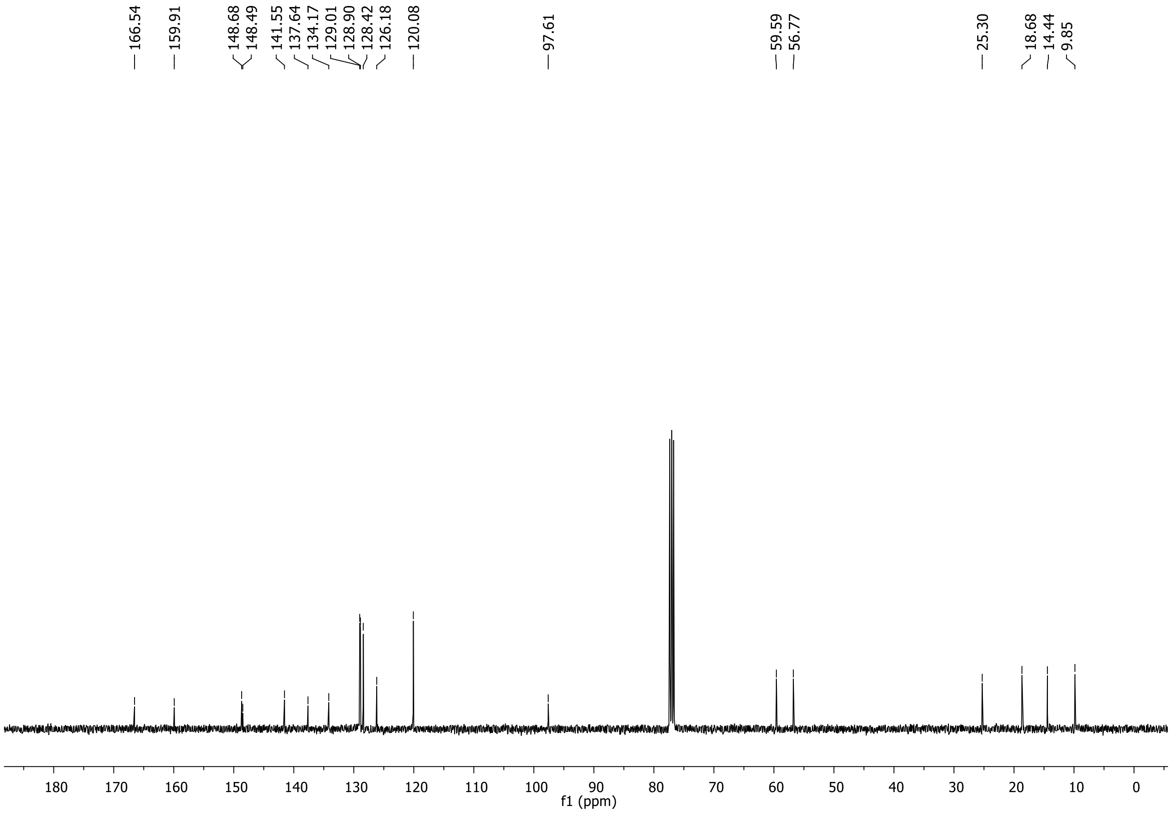

Ethyl 3-ethyl-5-(4-methoxyphenyl)-7-methyl-1-phenyl-1,5-dihydro-[1,2,4]triazolo[4,3-*a*]pyrimidine-6-carboxylate 4j

Figure S17: ^{1}H NMR (400 MHz, $CDCl_3$) spectra of **4j**.

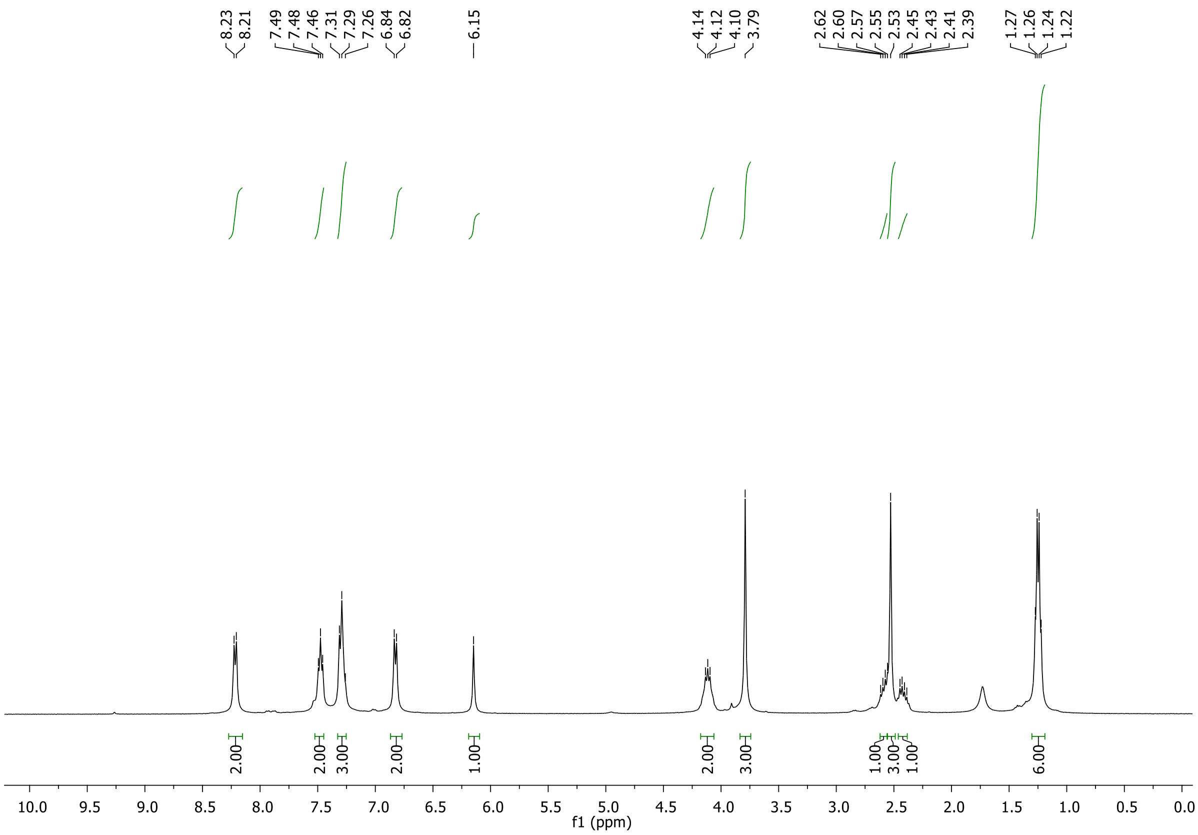

Figure S18: ^{13}C NMR (100 MHz, $CDCl_3$) spectra of **4j**.

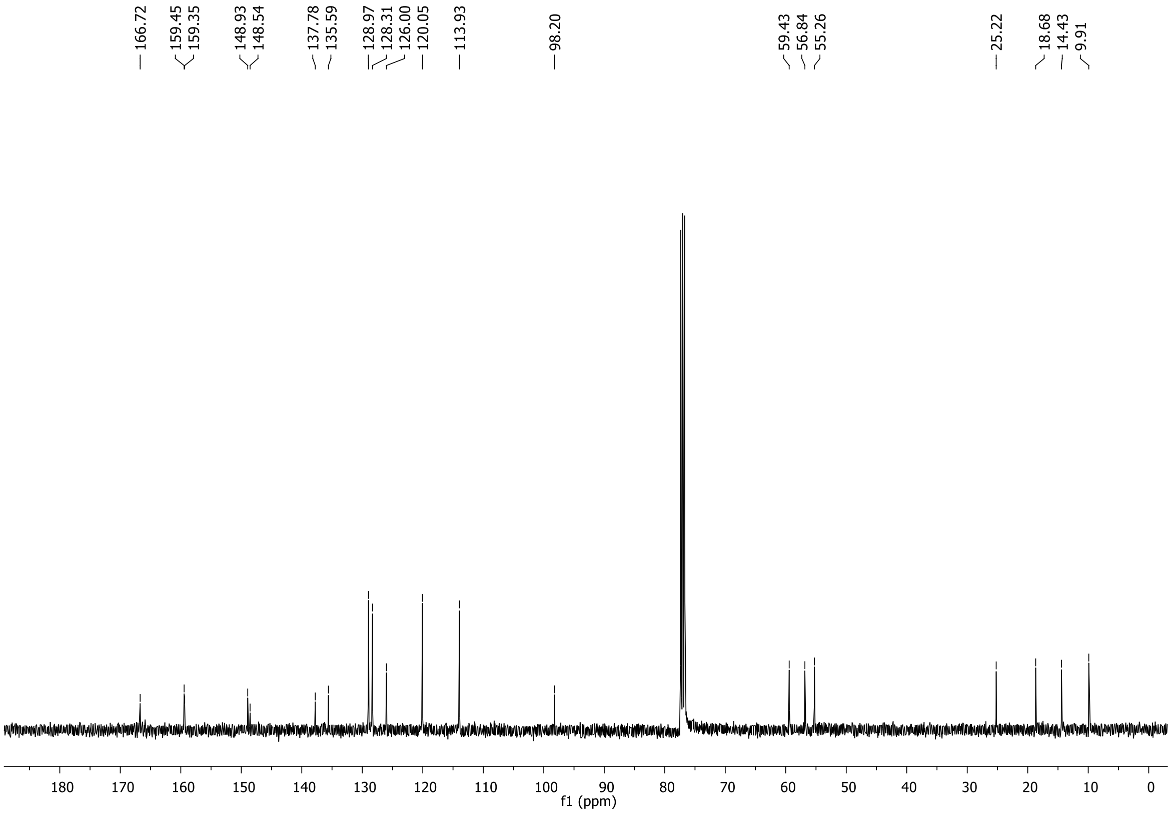

Ethyl 3-ethyl-7-methyl-1-phenyl-5-(*p*-tolyl)-1,5-dihydro-[1,2,4]triazolo[4,3-*a*]pyrimidine-6-carboxylate 4k

Figure S19: ^{1}H NMR (400 MHz, $CDCl_3$) spectra of **4k**.

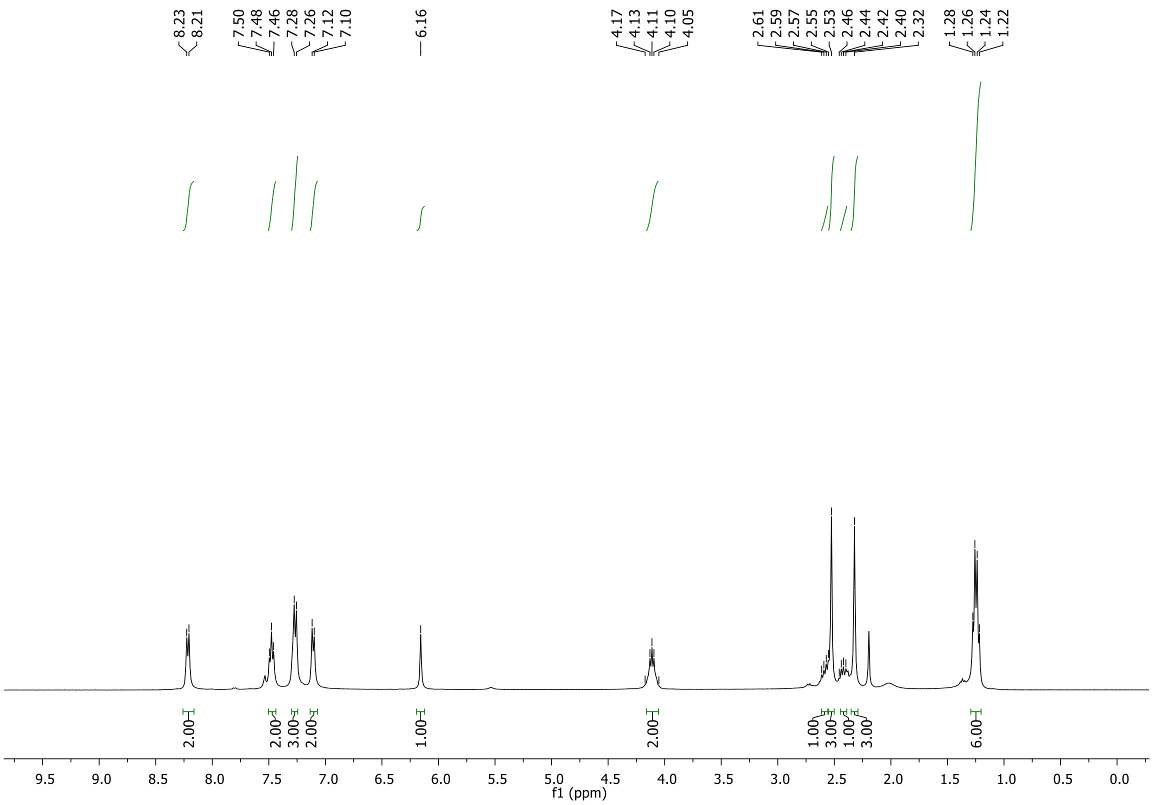

Figure S20: ^{13}C NMR (100 MHz, $CDCl_3$) spectra of **4k**.

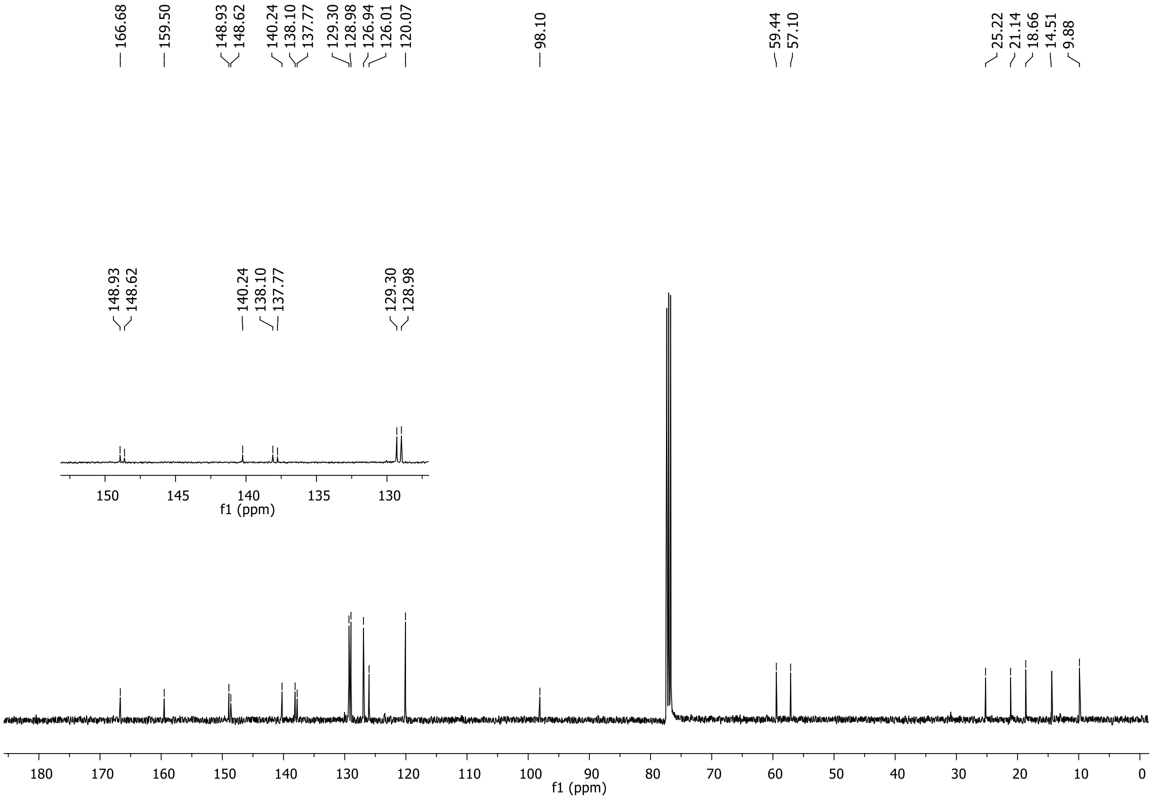

Ethyl 3-ethyl-7-methyl-5-(4-nitrophenyl)-1-phenyl-1,5-dihydro-[1,2,4]triazolo[4,3-*a*]pyrimidine-6-carboxylate 4l

Figure S21: ^{1}H NMR (400 MHz, $CDCl_3$) spectra of **4l**.

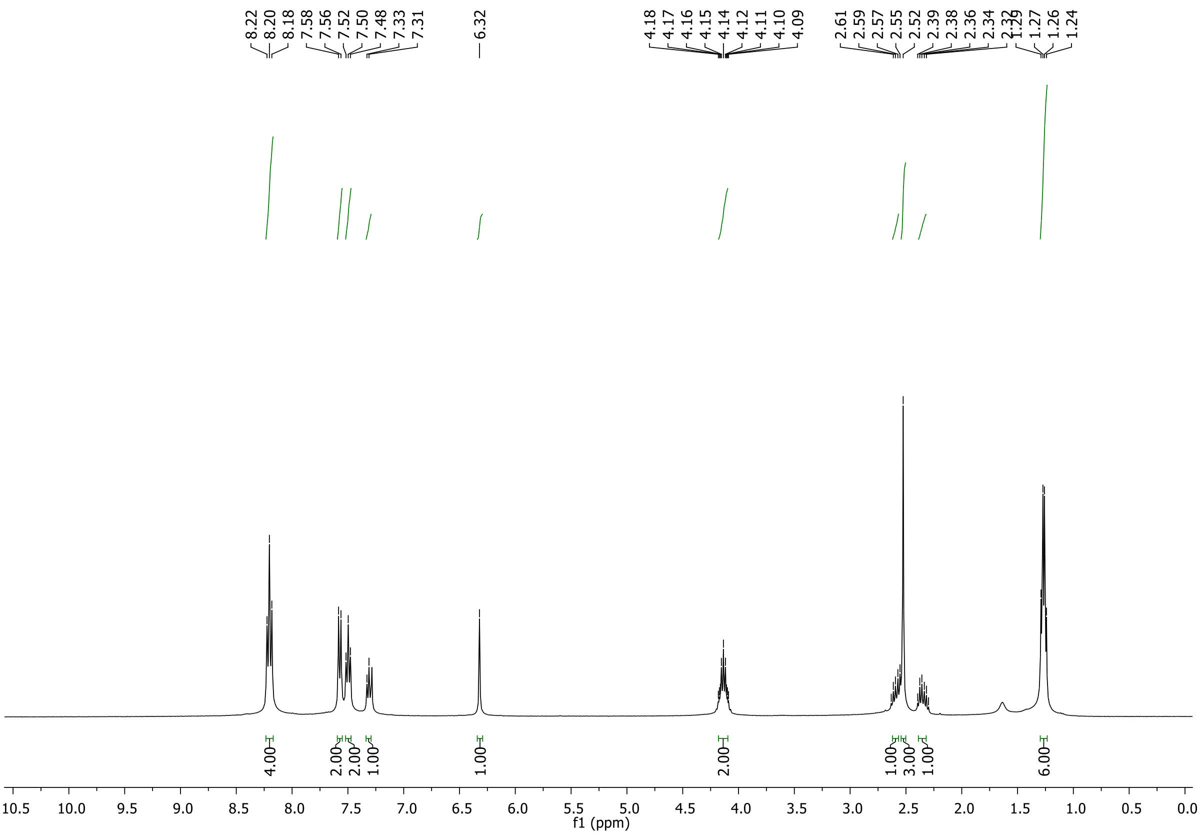

Figure S22: ^{13}C NMR (100 MHz, $CDCl_3$) of spectra **4l**.

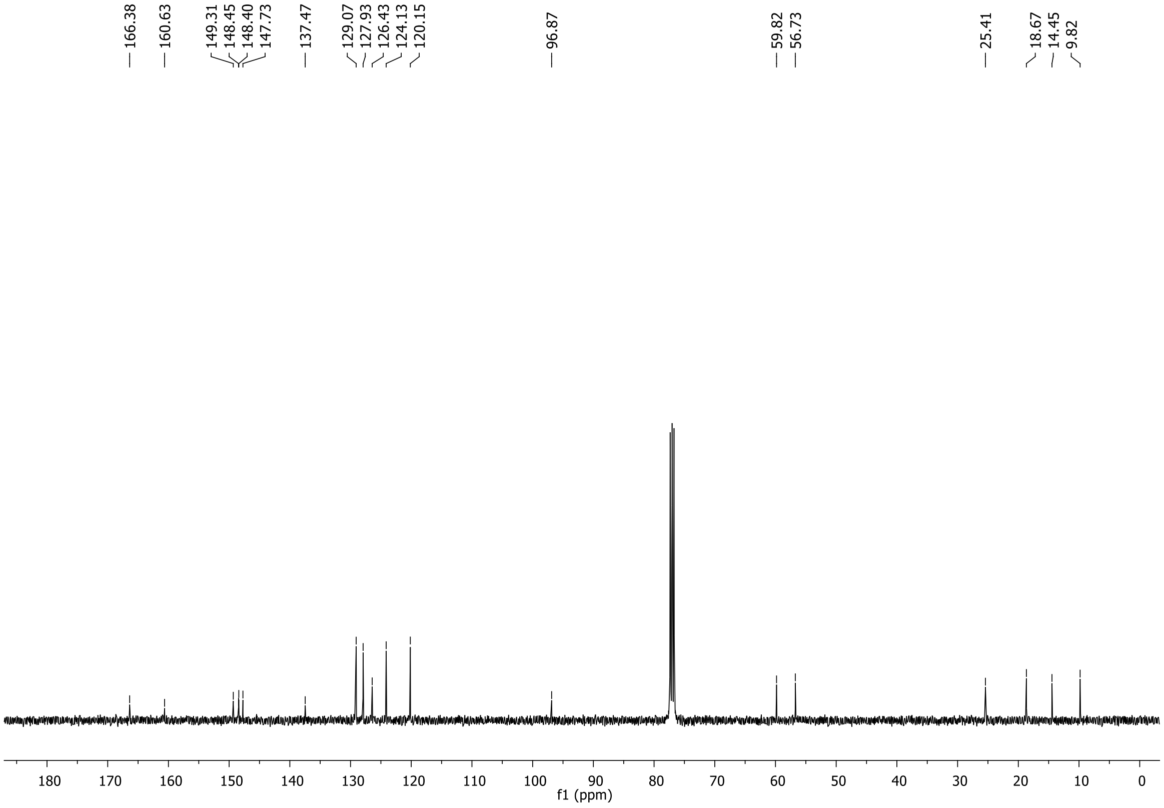

Ethyl 3-ethyl-5-(4-fluorophenyl)-7-methyl-1-phenyl-1,5-dihydro-[1,2,4]triazolo[4,3-*a*]pyrimidine-6-carboxylate 4m

Figure S23: ^{1}H NMR (400 MHz, $CDCl_3$) spectra of **4m**.

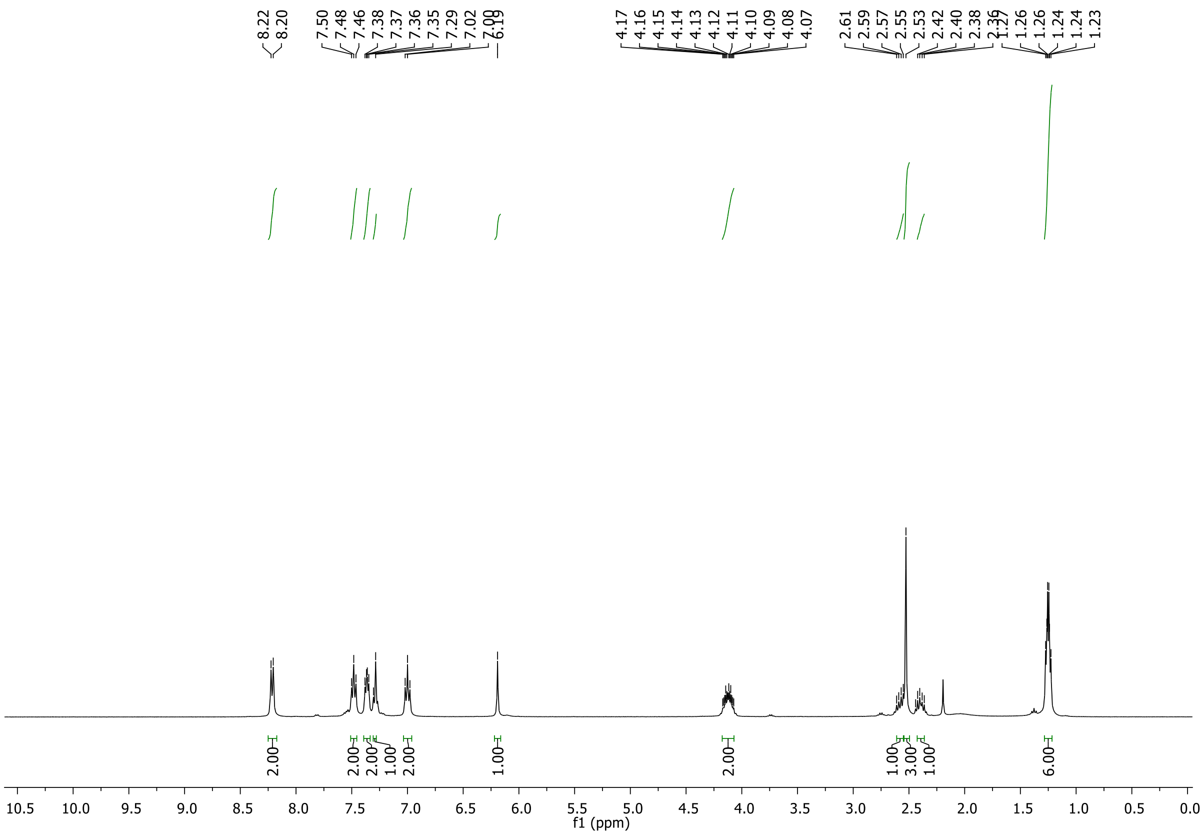

Figure S24: ^{13}C NMR (100 MHz, $CDCl_3$) spectra of **4m**.

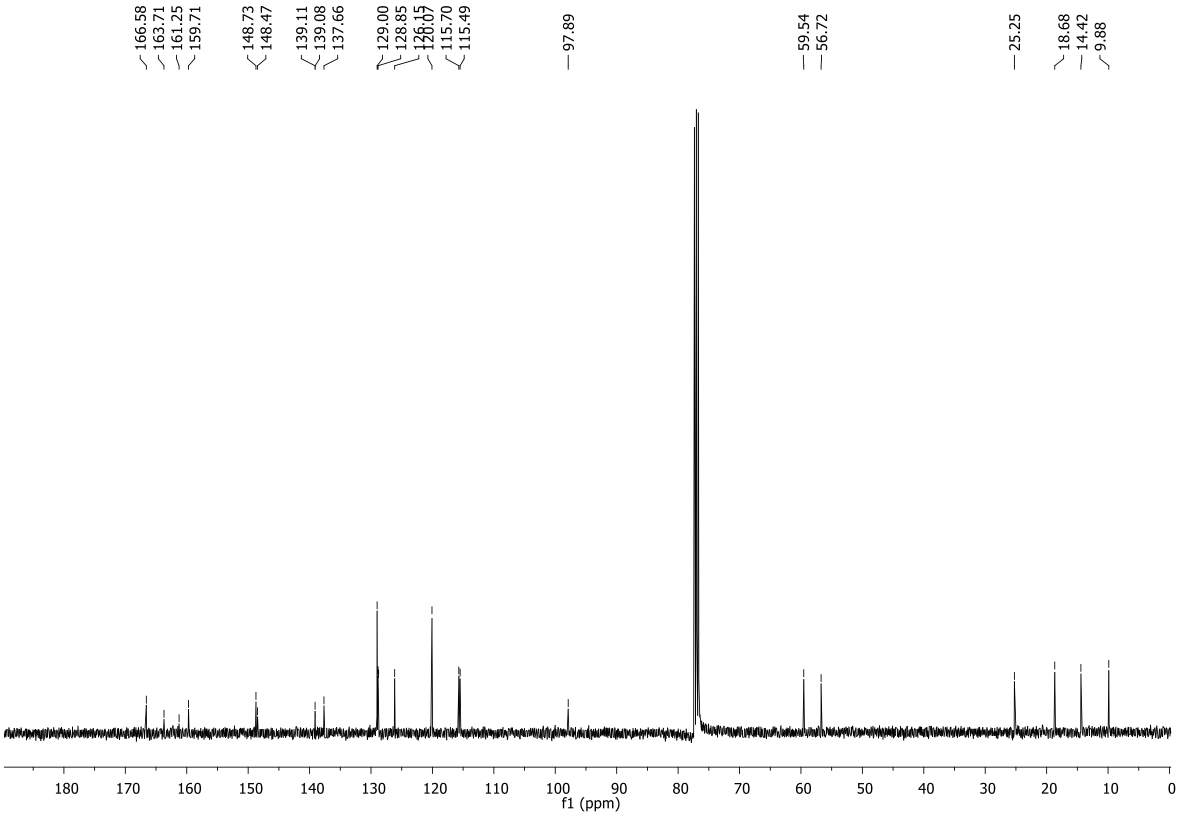

Ethyl 5-(2,4-dichlorophenyl)-3-ethyl-7-methyl-1-phenyl-1,5-dihydro-[1,2,4]triazolo[4,3-*a*]pyrimidine-6-carboxylate 4n

Figure S25: ^{1}H NMR (400 MHz, $CDCl_3$) spectra of **4n**.

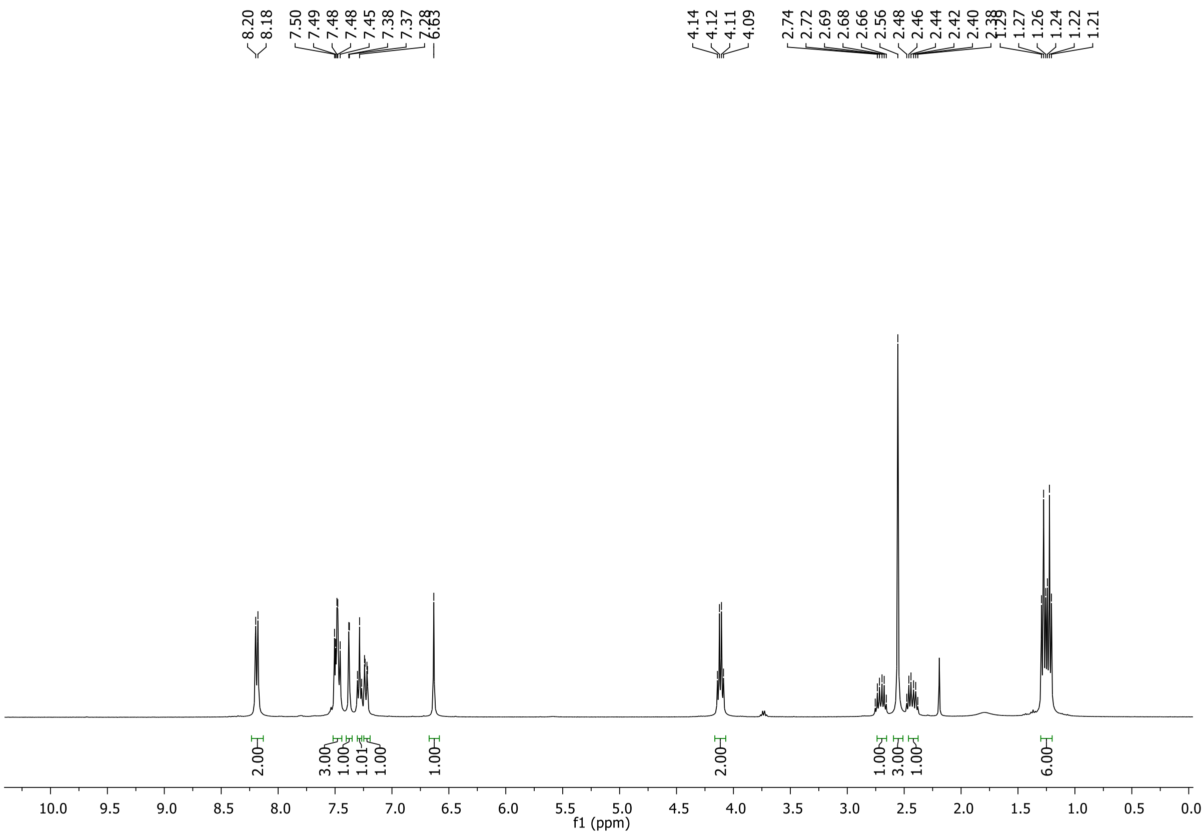

Figure S26: ^{13}C NMR (100 MHz, $CDCl_3$) spectra of **4n**.

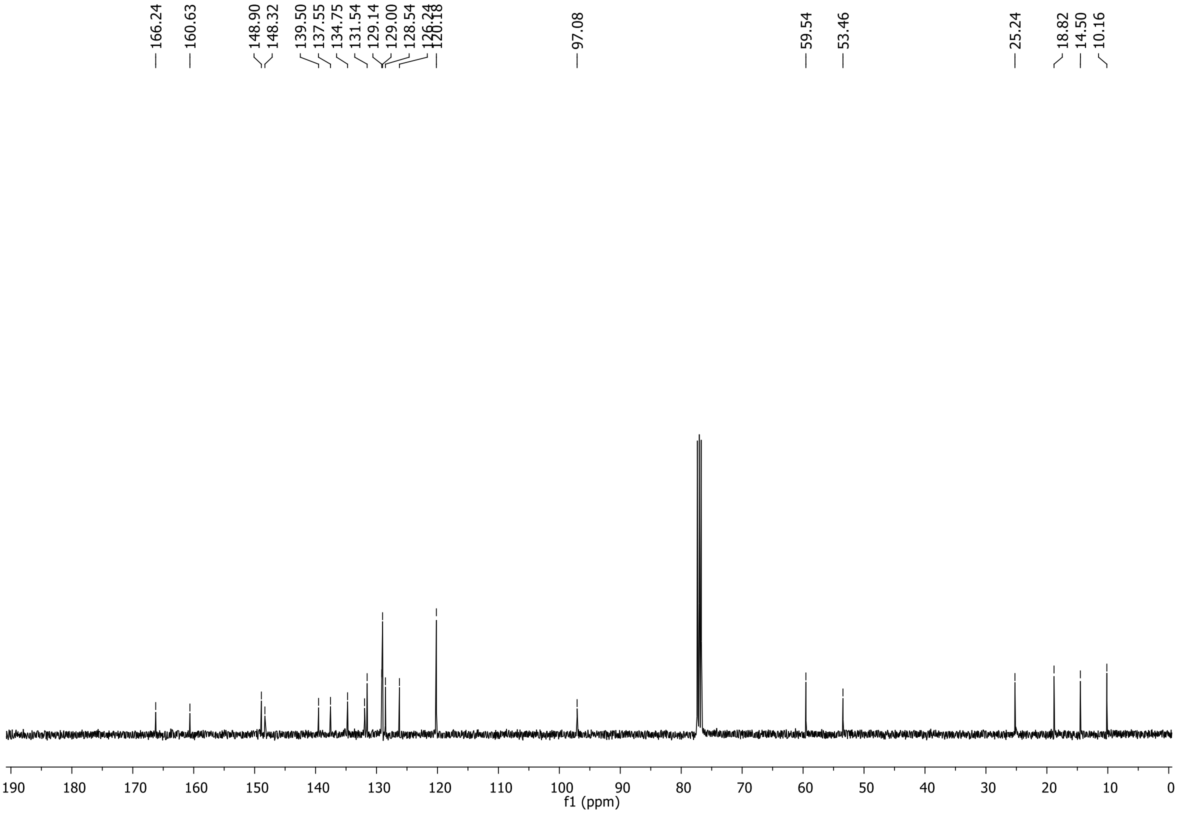

Figure S27: 2D NMR HMBC sequence of compound **4f**.

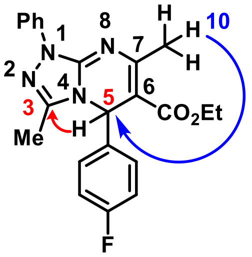

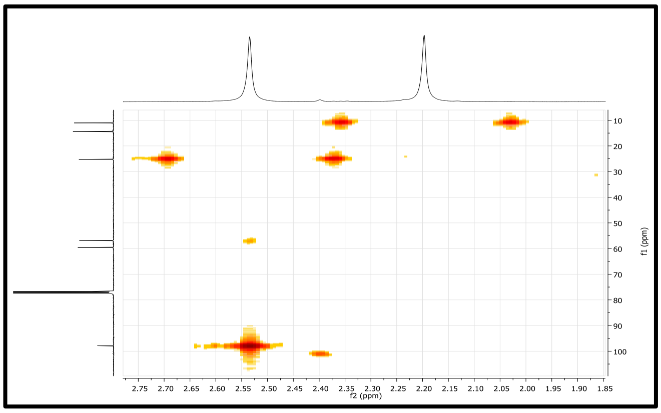

Correlation between C5 and H10

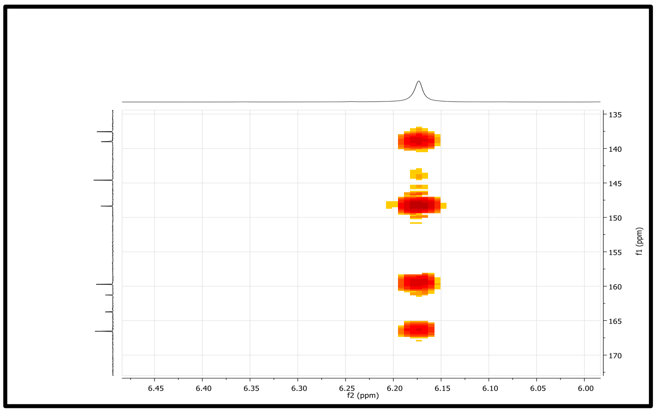

Correlation between C3 and H5

Figure S28: FT-IR spectrum of **4n**.

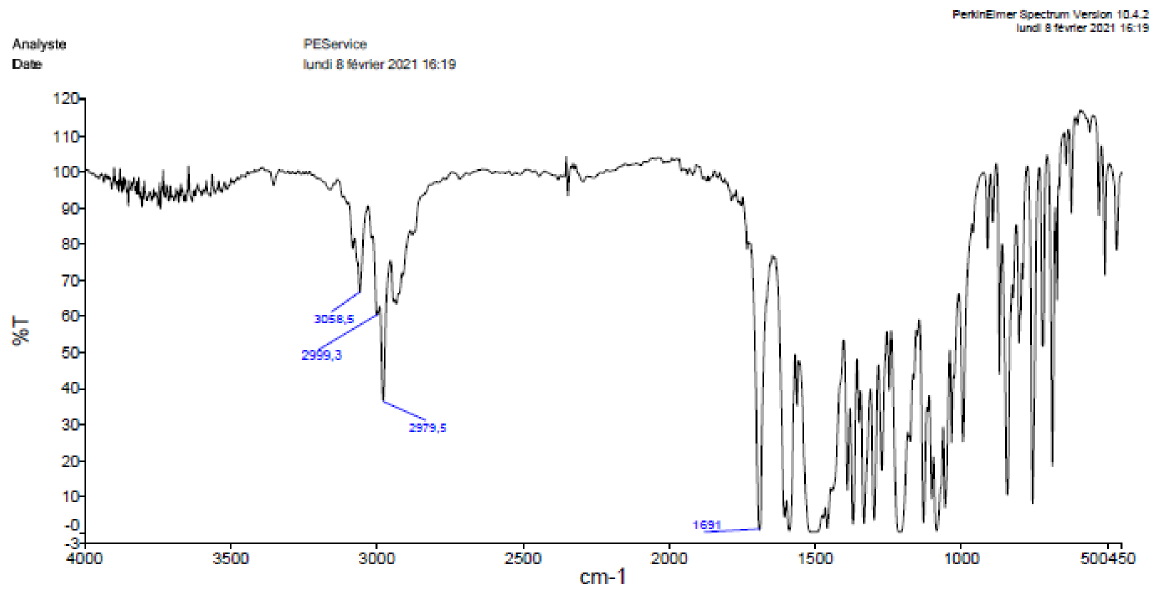

Contents

INFORMATIONS 19

COORDONNEES DU DEMANDEUR 19

DEMANDE D'ANALYSE 19

REMARQUES 19

PREPARATION DES ECHANTILLONS 19

ECHANTILLON 13A **4A** 19

ECHANTILLON 13B **4B** 20

ECHANTILLON 13C **4C** 20

ECHANTILLON 13D **4D** 21

ECHANTILLON 13E **4E** 21

ECHANTILLON 13F **4F** 22

ECHANTILLON 13G **4G** 22

ECHANTILLON 13H **4H** 23

ECHANTILLON 13I **4I** 23

ECHANTILLON 13J **4J** 24

ECHANTILLON 13K **4K** 24

ECHANTILLON 13L **4L** 25

ECHANTILLON 13M **4M** 25

ECHANTILLON 13N **4N** 26

TABLEAU RECAPITULATIF 26

Informations

Coordonnées du demandeur

Nom : Mohamed Abarbri

E-Mail : mohamed.abarbri@univ-tours.fr

Responsable : François Tran Van

Demande d'analyse

Demande d'analyse HRMS : confirmation de structure connue, 14 échantillons, série 1

Remarques

Analyses effectuées par : Antoine Lefevre et Frédéric Montigny
Interprétation des résultats et rapport d'analyse : Frédéric Montigny
Toutes les acquisitions HRMS ont été effectuées à une résolution de 140 000 pour une m/z de 200.
La détection se fait systématiquement dans les modes positif et négatif, par contre les résultats du mode négatif ne sont présentés que s'ils apportent des informations supplémentaires par rapport aux résultats du mode positif.

Préparation des échantillons

Tous les échantillons ont été préparés en solution dans MeOH /CH_2Cl_2 (70 /30).

Echantillon 13a **4a**

Indications fournies :

Masse moléculaire : 374.1743 g.mol^{-1}
Formule brute : $C_{22}H_{22}N_4O_2$

Détection mode positif :

On observe un pic majoritaire M+1 à 375.18117 (formule brute proposée $C_{22}H_{23}N_4O_2$ à -1.02985 ppm près, résolution supérieure à 100000) correspondant au composé recherché. La répartition isotopique expérimentale est cohérente avec la formule brute proposée.

Détection mode négatif :

Aucune information supplémentaire.

Conclusion:
La masse moléculaire obtenue et le massif isotopique permettent de confirmer la structure du composé analysé.

Echantillon 13b **4b**

Indications fournies :

Masse moléculaire : 408.1353 $g.mol^{-1}$
Formule brute : $C_{22}H_{21}ClN_4O_2$

Détection mode positif :

On observe deux pics majoritaires M+1 à 409.14243 (formule brute proposée $C_{22}H_{22}{}^{35}ClN_4O_2$ à -0.35937 ppm près, résolution supérieure à 100000) et M+1 à 411.13948 (formule brute proposée $C_{22}H_{22}{}^{37}ClN_4O_2$ à -0.35363 ppm près, résolution supérieure à 100000) correspondants tous deux au composé recherché. La répartition isotopique expérimentale est cohérente avec la formule brute proposée.

Détection mode négatif :

Aucune information supplémentaire.

Conclusion:
La masse moléculaire obtenue et le massif isotopique permettent de confirmer la structure du composé analysé.

Echantillon 13c **4c**

Indications fournies :

Masse moléculaire : 404.1848 $g.mol^{-1}$
Formule brute : $C_{23}H_{24}N_4O_3$

Détection mode positif :

On observe un pic majoritaire M+1 à 405.19201 (formule brute proposée $C_{23}H_{25}N_4O_3$ à -0.25852 ppm près, résolution supérieure à 100000) correspondant au composé recherché. La répartition isotopique expérimentale est cohérente avec la formule brute proposée.

Détection mode négatif :

Aucune information supplémentaire.

Conclusion:
La masse moléculaire obtenue et le massif isotopique permettent de confirmer la structure du composé analysé.

Echantillon 13d **4d**

Indications fournies :

Masse moléculaire : 388.1899 $g.mol^{-1}$
Formule brute : $C_{23}H_{24}N_4O_2$

Détection mode positif :

On observe un pic majoritaire M+1 à 389.19704 (formule brute proposée $C_{23}H_{25}N_4O_2$ à -0.40777 ppm près, résolution supérieure à 100000) correspondant au composé recherché. La répartition isotopique expérimentale est cohérente avec la formule brute proposée.

Détection mode négatif :

Aucune information supplémentaire.

Conclusion:

La masse moléculaire obtenue et le massif isotopique permettent de confirmer la structure du composé analysé.

Echantillon 13e **4e**

Indications fournies :

Masse moléculaire : 419.1594 $g.mol^{-1}$
Formule brute : $C_{22}H_{21}N_5O_4$

Détection mode positif :

On observe un pic majoritaire M+1 à 420.16642 (formule brute proposée $C_{22}H_{22}N_5O_4$ à -0.49883 ppm près, résolution supérieure à 100000) correspondant au composé recherché. La répartition isotopique expérimentale est cohérente avec la formule brute proposée.

Détection mode négatif :

Aucune information supplémentaire.

Conclusion:

La masse moléculaire obtenue et le massif isotopique permettent de confirmer la structure du composé analysé.

Echantillon 13f **4f**

Indications fournies :

Masse moléculaire : 392.1649 g.mol^{-1}
Formule brute : $C_{22}H_{21}FN_4O_2$

Détection mode positif :

On observe un pic majoritaire M+1 à 393.17188 (formule brute proposée $C_{22}H_{22}FN_4O_2$ à -0.64354 ppm près, résolution supérieure à 100000) correspondant au composé recherché. La répartition isotopique expérimentale est cohérente avec la formule brute proposée.

Détection mode négatif :

Aucune information supplémentaire.

Conclusion:

La masse moléculaire obtenue et le massif isotopique permettent de confirmer la structure du composé analysé.

Echantillon 13g **4g**

Indications fournies :

Masse moléculaire : 442.0963 g.mol^{-1}
Formule brute : $C_{22}H_{20}Cl_2N_4O_2$

Détection mode positif :

On observe trois pics majoritaires M+1 à 443.10342 (formule brute proposée $C_{22}H_{21}{}^{35}Cl_2N_4O_2$ à -0.41418 ppm près, résolution supérieure à 100000), M+1 à 445.10056 (formule brute proposée $C_{22}H_{21}{}^{35}Cl{}^{37}ClN_4O_2$ à -0.21925 ppm près, résolution supérieure à 100000) et M+1 à 447.09756 (formule brute proposée $C_{22}H_{21}{}^{37}Cl_2N_4O_2$ à -0.33835 ppm près, résolution supérieure à 90000) correspondants tous trois au composé recherché. La répartition isotopique expérimentale est cohérente avec la formule brute proposée.

Détection mode négatif :

Aucune information supplémentaire.

Conclusion:

La masse moléculaire obtenue et le massif isotopique permettent de confirmer la structure du composé analysé.

Echantillon 13h **4h**

Indications fournies :

Masse moléculaire : 388.1899 g.mol^{-1}
Formule brute : $C_{23}H_{24}N_4O_2$

Détection mode positif :

On observe un pic majoritaire M+1 à 389.19701 (formule brute proposée $C_{23}H_{25}N_4O_2$ à -0.50543 ppm près, résolution supérieure à 100000) correspondant au composé recherché. La répartition isotopique expérimentale est cohérente avec la formule brute proposée.

Détection mode négatif :

Aucune information supplémentaire.

Conclusion:

La masse moléculaire obtenue et le massif isotopique permettent de confirmer la structure du composé analysé.

Echantillon 13i **4i**

Indications fournies :

Masse moléculaire : 422.1510 g.mol^{-1}
Formule brute : $C_{23}H_{23}ClN_4O_2$

Détection mode positif :

On observe deux pics majoritaires M+1 à 423.15818 (formule brute proposée $C_{23}H_{24}{}^{35}ClN_4O_2$ à -0.12936 ppm près, résolution supérieure à 100000) et M+1 à 425.15519 (formule brute proposée $C_{23}H_{24}{}^{37}ClN_4O_2$ à -0.20957 ppm près, résolution supérieure à 100000) correspondants tous deux au composé recherché. La répartition isotopique expérimentale est cohérente avec la formule brute proposée.

Détection mode négatif :

Aucune information supplémentaire.

Conclusion:

La masse moléculaire obtenue et le massif isotopique permettent de confirmer la structure du composé analysé.

Echantillon 13j **4j**

Indications fournies :

Masse moléculaire : 418.2005 g.mol^{-1}
Formule brute : $C_{24}H_{26}N_4O_3$

Détection mode positif :

On observe un pic majoritaire M+1 à 419.20754 (formule brute proposée $C_{24}H_{27}N_4O_3$ à -0.53729 ppm près, résolution supérieure à 100000) correspondant au composé recherché. La répartition isotopique expérimentale est cohérente avec la formule brute proposée.

Détection mode négatif :

Aucune information supplémentaire.

Conclusion:

La masse moléculaire obtenue et le massif isotopique permettent de confirmer la structure du composé analysé.

Echantillon 13k **4k**

Indications fournies :

Masse moléculaire : 402.2056 g.mol^{-1}
Formule brute : $C_{24}H_{26}N_4O_2$

Détection mode positif :

On observe un pic majoritaire M+1 à 403.21270 (formule brute proposée $C_{24}H_{27}N_4O_2$ à -0.36721 ppm près, résolution supérieure à 100000) correspondant au composé recherché. La répartition isotopique expérimentale est cohérente avec la formule brute proposée.

Détection mode négatif :

Aucune information supplémentaire.

Conclusion:

La masse moléculaire obtenue et le massif isotopique permettent de confirmer la structure du composé analysé.

Echantillon 13l **4l**

Indications fournies :

Masse moléculaire : 433.1750 g.mol^{-1}
Formule brute : $C_{23}H_{23}N_{5}O_{4}$

Détection mode positif :

On observe un pic faible M+1 à 434.18204 (formule brute proposée $C_{23}H_{24}N_{5}O_{4}$ à -0.55194 ppm près, résolution supérieure à 90000) correspondant au composé recherché. La répartition isotopique expérimentale est cohérente avec la formule brute proposée.

Détection mode négatif :

Aucune information supplémentaire.

Conclusion:

La masse moléculaire obtenue et le massif isotopique permettent de confirmer la structure du composé analysé.

Echantillon 13m **4m**

Indications fournies :

Masse moléculaire : 406.1805 g.mol^{-1}
Formule brute : $C_{23}H_{23}FN_{4}O_{2}$

Détection mode positif :

On observe un pic majoritaire M+1 à 407.18772 (formule brute proposée $C_{23}H_{24}FN_{4}O_{2}$ à -0.14086 ppm près, résolution supérieure à 100000) correspondant au composé recherché. La répartition isotopique expérimentale est cohérente avec la formule brute proposée.

Détection mode négatif :

Aucune information supplémentaire.

Conclusion:
La masse moléculaire obtenue et le massif isotopique permettent de confirmer la structure du composé analysé.

Echantillon 13n **4n**

Indications fournies :

Masse moléculaire : 456.1120 $g.mol^{-1}$
Formule brute : $C_{23}H_{22}Cl_2N_4O_2$

Détection mode positif :

On observe trois pics majoritaires M+1 à 457.11927 (formule brute proposée $C_{23}H_{23}{}^{35}Cl_2N_4O_2$ à 0.01816 ppm près, résolution supérieure à 100000), M+1 à 459.11643 (formule brute proposée $C_{23}H_{23}{}^{35}Cl^{37}ClN_4O_2$ à 0.26628 ppm près, résolution supérieure à 100000) et M+1 à 461.11352 (formule brute proposée $C_{23}H_{23}{}^{37}Cl_2N_4O_2$ à 0.34527 ppm près, résolution supérieure à 90000) correspondants tous trois au composé recherché. La répartition isotopique expérimentale est cohérente avec la formule brute proposée.

Détection mode négatif :

Aucune information supplémentaire.

Conclusion:

La masse moléculaire obtenue et le massif isotopique permettent de confirmer la structure du composé analysé.

Tableau récapitulatif

Bilan :

Nom	Ref.	Formule	M + 1 Calc.	M+ 1 Exp.	ppm	M - 1 Calc.	Validation
13a	**4a**	$C_{22}H_{22}N_4O_2$	375.18155	375.18117	-1.02985	373.16700	**Oui**
13b	**4b**	$C_{22}H_{21}{}^{35}ClN_4O_2$<br>$C_{22}H_{21}{}^{37}ClN_4O_2$	409.14258<br>411.13963	409.14243<br>411.13948	-0.35937<br>-0.35363	407.12803<br>409.12508	**Oui**
13c	**4c**	$C_{23}H_{24}N_4O_3$	405.19212	405.19201	-0.25852	403.17756	**Oui**
13d	**4d**	$C_{23}H_{24}N_4O_2$	389.19720	389.19704	-0.40777	387.18265	**Oui**
13e	**4e**	$C_{22}H_{21}N_5O_4$	420.16663	420.16642	-0.49883	418.15208	**Oui**
13f	**4f**	$C_{22}H_{21}FN_4O_2$	393.17213	393.17188	-0.64354	391.15758	**Oui**
13g	**4g**	$C_{22}H_{20}{}^{35}Cl_2N_4O_2$<br>$C_{22}H_{20}{}^{35}Cl^{37}ClN_4O_2$<br>$C_{22}H_{20}{}^{37}Cl_2N_4O_2$	443.10361<br>445.10066<br>447.09771	443.10342<br>445.10056<br>447.09756	-0.41418<br>-0.21925<br>-0.33824	441.08905<br>443.08610<br>445.083154	**Oui**
13h	**4h**	$C_{23}H_{24}N_4O_2$	389.19720	389.19701	-0.50543	387.18265	**Oui**
13i	**4i**	$C_{23}H_{23}{}^{35}ClN_4O_2$<br>$C_{23}H_{23}{}^{37}ClN_4O_2$	423.15823<br>425.15528	423.15818<br>425.15519	-0.12936<br>-0.20957	421.14368<br>423.14073	**Oui**
13j	**4j**	$C_{24}H_{26}N_4O_3$	419.20777	419.20754	-0.53729	417.19321	**Oui**

13k	**4k**	$C_{24}H_{26}N_4O_2$	403.21285	403.21270	-0.36721	401.19830	**Oui**
13l	**4l**	$C_{23}H_{23}N_5O_4$	434.18228	434.18204	-0.55194	432.16773	**Oui**
13m	**4m**	$C_{23}H_{23}FN_4O_2$	407.18778	407.18772	-0.14086	405.17323	**Oui**
13n	**4n**	$C_{23}H_{22}{}^{35}Cl_2N_4O_2$<br>$C_{23}H_{22}{}^{35}Cl^{37}ClN_4O_2$<br>$C_{23}H_{22}{}^{37}Cl_2N_4O_2$	457.11926<br>459.11631<br>461.11336	457.11927<br>459.11643<br>464.11352	0.01816<br>0.26628<br>0.34527	455.10470<br>457.10175<br>459.09880	**Oui**
